# Mechanism-based tuning of insect 3,4-dihydroxyphenylacetaldehyde synthase for synthetic bioproduction of benzylisoquinoline alkaloids

**DOI:** 10.1038/s41467-019-09610-2

**Published:** 2019-05-01

**Authors:** Christopher J. Vavricka, Takanobu Yoshida, Yuki Kuriya, Shunsuke Takahashi, Teppei Ogawa, Fumie Ono, Kazuko Agari, Hiromasa Kiyota, Jianyong Li, Jun Ishii, Kenji Tsuge, Hiromichi Minami, Michihiro Araki, Tomohisa Hasunuma, Akihiko Kondo

**Affiliations:** 1 Graduate School of Science, Technology and Innovation, Kobe University, 1-1 Rokkodai-cho, Nada-ku, Kobe, 657-8501 Japan; 2 Mitsui Knowledge Industry Co., Ltd. (MKI), 2-3-33 Nakanoshima, Kita-ku, Osaka, 530-0005 Japan; 3 Graduate School of Medicine, Kyoto University, Yoshida-Konoe-cho, Sakyo-ku, Kyoto, 606-8501 Japan; 4 Graduate School of Environmental and Life Science, Okayama University, 1-1-1, Tsushima-Naka, Kita-ku, Okayama, 700-8530 Japan; 5 Department of Biochemistry, Virginia Polytechnic and State University, 111 Engel Hall, Mail Code: 0308, Blacksburg, VA 24061 USA; 6 Research Institute for Bioresources and Biotechnology, Ishikawa Prefectural University, 1-308, Suematsu, Nonoichi-shi, Ishikawa-ken, 921-8836 Japan; 7 Engineering Biology Research Center, Kobe University, 1-1 Rokkodai-cho, Nada-ku, Kobe, 657-8501 Japan; 8 Department of Chemical Science and Engineering, Graduate School of Engineering, 1-1 Rokkodai-cho, Nada-ku, Kobe, 657-8501 Japan

**Keywords:** Enzyme mechanisms, Metabolic engineering, Applied microbiology, Synthetic biology

## Abstract

Previous studies have utilized monoamine oxidase (MAO) and L-3,4-dihydroxyphenylalanine decarboxylase (DDC) for microbe-based production of tetrahydropapaveroline (THP), a benzylisoquinoline alkaloid (BIA) precursor to opioid analgesics. In the current study, a phylogenetically distinct *Bombyx mori* 3,4-dihydroxyphenylacetaldehyde synthase (DHPAAS) is identified to bypass MAO and DDC for direct production of 3,4-dihydroxyphenylacetaldehyde (DHPAA) from L-3,4-dihydroxyphenylalanine (L-DOPA). Structure-based enzyme engineering of DHPAAS results in bifunctional switching between aldehyde synthase and decarboxylase activities. Output of dopamine and DHPAA products is fine-tuned by engineered DHPAAS variants with Phe79Tyr, Tyr80Phe and Asn192His catalytic substitutions. Balance of dopamine and DHPAA products enables improved THP biosynthesis via a symmetrical pathway in *Escherichia coli*. Rationally engineered insect DHPAAS produces (*R*,*S*)-THP in a single enzyme system directly from L-DOPA both in vitro and in vivo, at higher yields than that of the wild-type enzyme. However, DHPAAS-mediated downstream BIA production requires further improvement.

## Abstract (web summary)

Bioproduction of tetrahydropapaveroline (THP) is limited by the specificity of monoamine oxidase (MAO). Here, the authors identify an insect 3,4-dihydroxyphenylacetaldehyde synthase (DHPAAS) that can bypass MAO for direct aldehyde production and demonstrate bifunctional switching of DHPAAS for efficient THP production.

## Introduction

Recent progress in synthetic biology and metabolic engineering offers potential to optimize the bioproduction of virtually any compound^1–5^. Engineered metabolic systems are now being designed for production of biofuels, polymers, bulk chemicals, and pharmaceuticals^1,2^. Computational methods to select the best metabolic pathways from thousands of possibilities have been demonstrated for production of 1,4-butanediol^4^. The combination of metabolic engineering with enzyme engineering can even be used to construct artificial biosynthetic pathways to compounds including 2,4-dihydroxybutyric acid^5^.

With the goal of further developing enzyme engineering as a practical approach that can expand the scope of bioproduction targets, benzylisoquinoline alkaloid (BIA) biosynthesis is presented as an example for synthetic enzyme development. Opioid analgesics are derived from BIAs and are essential medicines for pain and palliative care as defined by the World Health Organization^6^. Microbial production of pharmaceutical alkaloids offers advantages in terms of cost efficiency, environmental sustainability, and process control^7^. Recently, bioproduction of opioid alkaloids has been achieved in yeast via a norcoclaurine (higenamine) containing pathway^8,9^ and in *Escherichia coli* via a tetrahydropapaveroline (THP, norlaudanosoline) containing pathway^10,11^. The THP pathway requires one less enzymatic step and has afforded the highest BIA titer of 1 mM THP (287 mg/L). However, current THP bioproduction relies upon monoamine oxidase (MAO), a membrane bound flavoenzyme that is active towards many monoamines in addition to dopamine^12,13^. It is therefore desirable to engineer a soluble enzyme to improve production of THP, and downstream alkaloids including thebaine, which has been recently reported with microbial titers lower than 10 mg per litre^11^.

Utilizing BIA bioproduction as a model pathway, the current system aims to search for alternative enzyme engineering targets and learn alternative biosynthetic pathways. This synthetic biology workflow includes enzyme selection and learning for synthetic pathway design via the recently developed M-path prediction software^14^. Specifically, a 3,4-dihydroxyphenylacetaldehyde synthase (DHPAAS) is identified from *Bombyx mori* and engineered to switch between two distinct activities for the direct production of THP in a symmetrical pathway. Existing reports have applied substrate specificity engineering to metabolic engineering^5^. In contrast, the current study applies functional enzyme engineering to the assembly of an alternative bioproduction pathway.

## Results

### Pathway design and enzyme selection

**Fig. 1:**
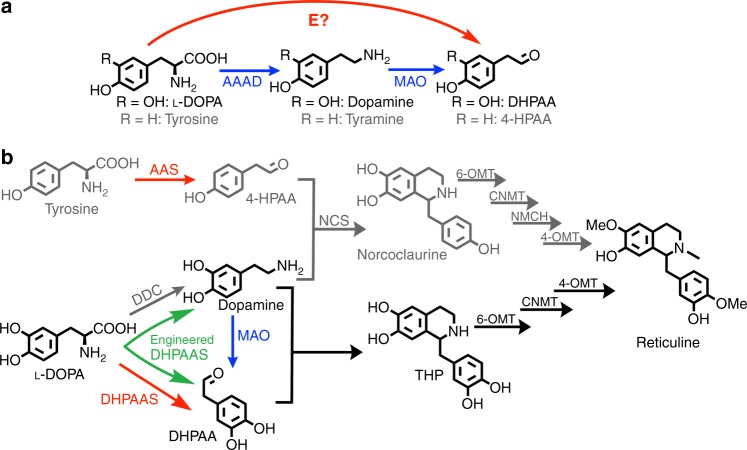
Design of a symmetrical THP pathway for reticuline bioproduction. **a** M-path enzyme (E) search identified phenylacetaldehyde synthase (PAAS), 4-HPAA Synthase (4-HPAAS), and DHPAAS as putative enzymes to directly produce 4-hydroxyphenylacetaldehyde (4-HPAA) from tyrosine or DHPAA from L-DOPA (Supplementary Table 1). **b** Multiple pathways lead to THP and norcoclaurine production, including a nonsymmetrical DDC-MAO-mediated pathway (blue and grey arrows), symmetrical DDC-DHPAAS/AAS-mediated pathways (red and grey arrows), and an engineered DHPAAS single enzyme system (green split arrows). (*S*)-norcoclaurine can be produced from 4-hydroxyphenylacetaldehyde (4-HPAA) and dopamine via the enzyme (*S*)-norcoclaurine synthase (NCS, EC 4.2.1.78). Dopamine and DHPAA undergo spontaneous Pictet-Spengler condensation to form THP (3-hydroxy-norcoclaurine), or this reaction can also be catalyzed by NCS. THP is converted to reticuline via norcoclaurine 6-*O*-methyltransferase (6-OMT), coclaurine *N*-methyltransferase (CNMT) and 3-hydroxy-*N*-methylcoclaurine 4-*O*-methyltransferase (4-OMT). An additional enzyme, *N*-methylcoclaurine 3-hydroxylase (NMCH), is necessary to produce reticuline from norcoclaurine. The 4-HPAA containing pathways are shown in grey as the current study focuses on the DHPAA containing pathway. All structures were drawn with ChemDraw

In 2014, the M-path computational platform was developed to predict putative metabolic pathways and enzymes that might catalyze new reactions^14^. M-path uses an iterative random algorithm to score chemical similarities and can be operated as a web-based tool. In contrast to searching known enzyme networks, M-path is advantageous in that it can predict unknown enzymatic reactions based upon substrate and product similarities. Furthermore, M-path can find reactions and pathways from a wide range of search space and easily expand the search space^14^. To explore alternative BIA production pathways from aromatic amino acids, the M-path search algorithm was tested. A combined database of updated enzyme entries from BRENDA (BRaunschweig ENzyme DAtabase)^15^ and Kyoto Encyclopedia of Genes and Genomes (KEGG)^16^ was used to increase enzyme targets. Aromatic aldehyde synthase (AAS) and DHPAAS were identified as putative shortcuts for production of 4-hydroxyphenylacetaldehyde (4-HPAA or 4-HPA) from L-tyrosine (Tyr), and 3,4-dihydroxyphenylacetaldehyde (DHPAA, DHPA or DOPAL) from L-3,4-dihydroxyphenylalanine (L-DOPA) (Fig. 1, Supplementary Table 1). Although authors were aware of the function of DHPAAS, this example of enzyme selection illustrates the importance of updating enzyme databases for prediction of recently characterized enzymes, as new functions are continuously discovered from nature and enzyme engineering^17^. The pairing of DHPAAS or AAS enzymes with 3,4-dihydroxyphenylalanine decarboxylase (DDC) results in symmetrical BIA bioproduction pathways that are distinct from the reported MAO-mediated pathways (Fig. 1b).

AAS and DHPAAS are recently established bifunctional enzymes, which catalyze the simultaneous decarboxylation and amino group oxidation of aromatic amino acids. Two enzymes discovered in plants, phenylacetaldehyde synthase (PAAS, EC 4.1.1.109)^18^ and 4-hydroxyphenylacetaldehyde synthase (4-HPAAS, EC 4.1.1.108)^19^, have been referred to as AAS. A more recently discovered enzyme from insects, DHPAAS (EC 4.1.1.107), catalyzes the oxidative decarboxylation of L-DOPA to produce DHPAA^20^, and this enzyme might therefore be considered as an AAS related protein. Phylogenetic analysis suggests that AAS and DHPAAS diverged from aromatic amino acid decarboxylase (AAAD, EC 4.1.1.28). Accordingly AAS, DHPAAS and AAAD are structurally similar and are all dependent upon pyridoxal 5**′-**phosphate (PLP) cofactor. Although AAS and DHPAAS were first assigned as EC 4.1.1.- by KEGG, many questions still remain about these recently characterized enzymes, which are not straightforward to classify due to bifunctional activities. Accordingly, EC 4.1.1.107–109 were only recently added to comprehensive enzymes databases like BRENDA, and rules for the selection of AAS or DHPAAS for bioproduction applications should be clearly established.

**Fig. 2:**
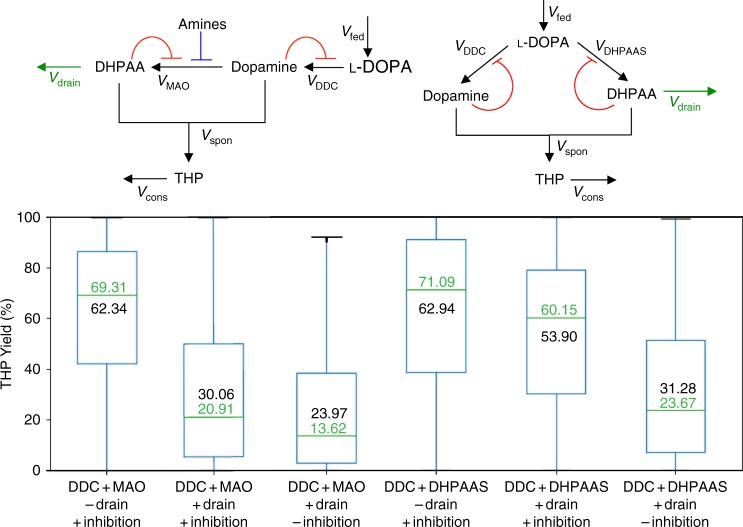
Predicted THP yields from DHPAAS- and MAO-mediated pathways. Conversion of L-DOPA to THP was simulated with and without DHPAA drain (green arrows), and also with and without product feedback inhibition (red flat-headed curves). The blue flat-headed line shows MAO inhibition by amines included in all MAO models. Green center lines show the median values (fiftieth percentile or second quartile), and the blue boxes contain the twenty-fifth percentile (first quartile) to seventy-fifth percentile (third quartile) of each dataset. The black whiskers show minimum and maximum values of each dataset. Median predicted THP yields are listed in green and mean predicted THP yields are listed in black within each box. All statistics were derived from *n* = 10,000 independent Monte–Carlo simulations

The construction of symmetrical AAS- and DHPAAS-mediated BIA production pathways may offer some advantages over the corresponding nonsymmetrical MAO-mediated pathways (Fig. 1). This includes increased specificity of soluble DHPAAS for L-DOPA relative to the specificity of MAO for dopamine. Mathematical models and numerical simulations were employed to compare the production of THP via the nonsymmetrical and symmetrical pathways (Supplementary Figures 1–3, Supplementary Table 2). Monte–Carlo simulation was performed with randomly generated values for parameters lacking reported data as described in Supplementary Methods and Supplementary Table 2. Later in vitro and in vivo tests suggested that highly reactive DHPAA may be depleted by chemical or enzymatic side-reactions. Including the carbon loss of DHPAA as ‘drain’ in dynamic models resulted in slightly lower THP yields (Fig. 2), indicating a better match to experimental levels. However, many diverse variables, including growth medium composition, pH, temperature, potential inhibitors, cofactor recycling, genetic regulation, and metabolic flux, should also be considered as learning data for the improvement of THP production. When factoring in product feedback inhibition^21^ together with DHPAA drain, symmetrical DDC-DHPAAS pathways produced higher predicted yields of THP than those of MAO-mediated pathways (Fig. 2, Supplementary Figure 3). These models suggest that DHPAAS-mediated pathways might offer clues to produce THP at levels higher than the best reported MAO-mediated THP benchmark of 1 mM (287 mg/L)^10^. Moreover, the improved performance of the feedback inhibition models emphasizes that the balance of dopamine and DHPAA is critical for optimal THP production. Therefore tuning the production of both DHPAA and dopamine by DHPAAS was pursued further.

### Enzyme design and structure-based engineering of DHPAAS

**Fig. 3:**
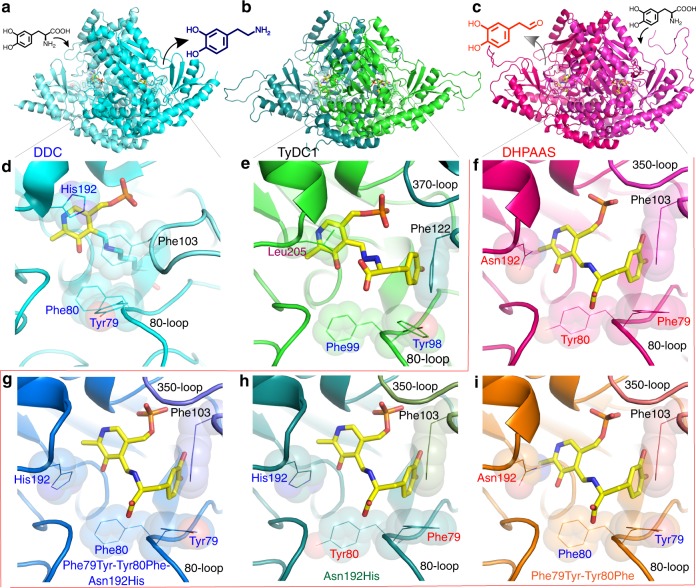
Structural analysis of AAAD and DHPAAS. Comparison of *Drosophila melanogaster* DDC in complex with PLP (cyan, **a** and **d**, PDB ID: 3K40)^25^, *P. somniferum* TyDC1 in complex with PLP-tyrosine (green, **b** and **e**), and wild-type *B. mori* DHPAAS in complex with PLP-DOPA (magenta, **c** and **f**). Phe79 and Tyr80 are conserved in DHPAAS sequences, whereas this motif is reversed to Tyr79 and Phe80 in typical AAAD, which includes DDC. In the *D. melanogaster* DDC-PLP complex with no substrate, Phe103 extends inwards towards the PLP cofactor. The bottom three panels show engineered active sites of Phe79Tyr-Tyr80Phe-Asn192His (**g**, blue), Asn192His (**h**, dark green) and Phe79Tyr-Tyr80Phe (**i**, orange) variants of *B. mori* DHPAAS, all in complex with PLP-DOPA

Comparison of putative plant AAS and insect DHPAAS structures was performed to rationally select optimal enzyme sequences for BIA production. Dimeric homology models of putative AAS and DHPAAS in complex with aromatic amino acid substrate covalently linked to PLP cofactor were generated with MODELLER^22^ operated within Chimera^23^ using default settings, followed by structure refinement with MOE (Fig. 3).

M-path identified 4-HPAAS as an enzyme to produce 4-HPAA, a key intermediate in plant BIA synthesis. Accordingly, we hypothesized that *P. somniferum* might utilize AAS activity for natural 4-HPAA bioproduction and explored *P. somniferum* sequences as potential AAS enzymes. Interestingly, *P. somniferum* tyrosine decarboxylase 1 (TyDC1), which was modeled based on the structure of *Sus Scrofa* DDC in complex with carbidopa (PDB ID: 1JS3)^24^, contains a leucine residue at the position corresponding to AAAD active site His192 (Fig. 3d, e), bringing attention to the 192 position as an important catalytic residue. However, aside from the unique TyDC1 Leu205, all *P. somniferum* TyDC sequences highly resemble that of canonical AAAD. In contrast, additional active site differences are observed when comparing putative insect DHPAAS sequences (Fig. 3). Therefore, focus was shifted towards insect DHPAAS for the selection of an optimal BIA bioproduction enzyme.

**Fig. 4:**
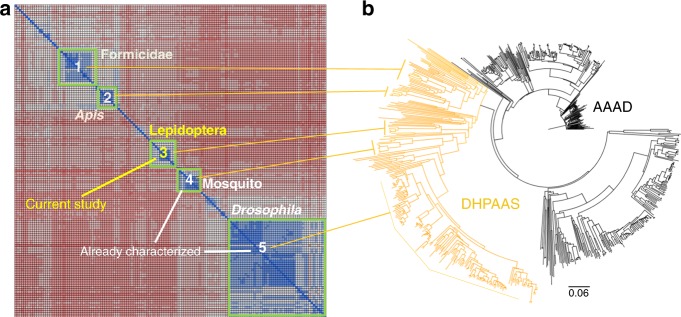
Phylogenetic classification of insect DHPAAS sequences. **a** Sequence similarity matrix of identified DHPAAS sequences which cluster into five distinct groups. **b** Phylogenetic tree of AAAD and related DHPAAS sequences. Supplementary Table 3 compares key DHPAAS and DDC active site residues

Many questions still remain about the evolution of insect DHPAAS, and its oxidative decarboxylation mechanism including the elucidation of all essential catalytic residues. In order to help clarify these questions and gain insight into mechanism-based engineering of DHPAAS, phylogenetic classification was carried out in combination with structural analysis. Phylogenetic analysis of 738 insect AAAD-related sequences resulted in the identification of 247 putative DHPAAS sequences and five distinct DHPAAS groups (Fig. 4, Supplementary Table 3).

Uncharacterized Lepidoptera DHPAAS comprises the central phylogenetic group (Fig. 4), and was selected as a candidate for DHPAAS mechanism studies. When analyzing insect DHPAAS, the unique region formed by residues Gly353 to Arg324 could not be well modeled using the structure of *D. melanogaster* DDC (PDB ID: 3K40)^25^ as the template. The structure of this 320–350-loop region, which participates in cross-dimer active site formation and substrate binding, improved when using a template of human histidine decarboxylase in complex with histidine methyl ester (PDB ID: 4E1O)^26^.

Comparison of DDC and DHPAAS active sites indicates that residue 192 (*B. mori* and *D. melanogaster* DHPAAS numbering) determines catalytic activity as that of a decarboxylase or aldehyde synthase (Fig. 3 and Supplementary Table 3). This 192 residue can form a hydrogen bond with the PLP-aromatic amino acid external aldimine, which undergoes oxidation in the DHPAAS mechanism. *Aedes aegypti* and *D. melanogaster* DHPAAS have been reported with Asn192 residues^20,27^. However, during the course of the current study, Asn192 was independently identified and verified as the key catalytic residue via structural and functional analysis.

Careful comparison of DDC and DHPAAS structures suggests that DHPAAS residues Phe79 and Tyr80 play an additional role in distinguishing DHPAAS activity from DDC activity (Figs 3 and 4, Supplementary Table 3). Tyr79-Phe80 is conserved in insect DDC, however this 79–80 motif is commonly reversed as Phe79-Tyr80 in insect DHPAAS, and these residues also surround the external aldimine of the PLP-substrate complex (Fig. 3). Therefore we hypothesized that these aromatic residues may also be involved in the DHPAAS catalytic mechanism and should be useful for the classification of DHPAAS. Within the five identified DHPAAS groups, Phe79-Tyr80 is conserved in *Apis* and mosquito. In *Drosophila* DHPAAS sequences annotated as isoform X1, Phe79-Tyr80 is conserved, whereas in those annotated as isoform X2 (includes NP_476592.1^27^), Tyr79-Tyr80 is conserved. Lepidoptera and formicidae DHPAAS groups contain a mixture of Phe79-Tyr80, Tyr79-Tyr80 and Tyr79-Phe80.

In this study, the *B. mori* sequence XM_004930959.2 was selected as a distinct DHPAAS sequence containing all three identified DHPAAS specific residues Phe79, Tyr80 and Asn192, as well as Gly353, which is reported to increase substrate specificity to L-DOPA^26^. In addition, Phe79Tyr, Tyr80Phe and Asn192His DHPAAS catalytic variants were designed to explore tuning the bifunctional production of dopamine and DHPAA.

### Switching the mechanism of DHPAAS

The native sequence of wild-type *B. mori XM_004930959.2* was synthesized and cloned into the *E. coli* expression vector pE-SUMO. DHPAAS was purified by metal affinity chromatography according to the methods section. DHPAAS activity was screened by separation of substrates and products using thin-layer chromatography (TLC). Enzymatic products were verified by LC-MS operated in multiple reaction monitoring (MRM) mode.

**Fig. 5:**
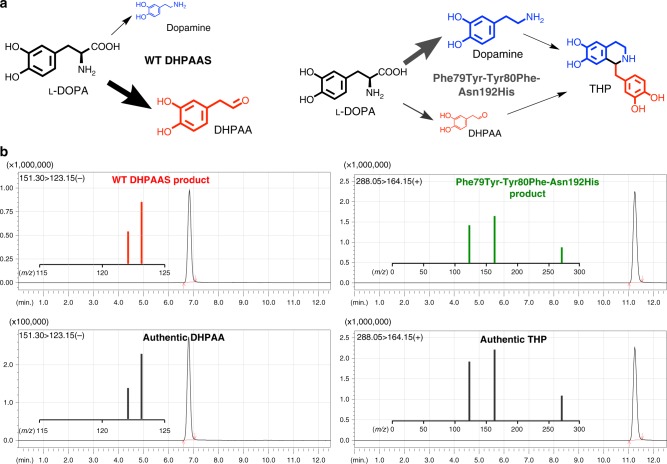
Mechanism switching of *B. mori* DHPAAS. **a** Comparison of wild-type *B. mori* DHPAAS and Phe79Tyr-Tyr80Phe-Asn192His DHPAAS reaction schemes. General product amounts are indicated by the relative size of each molecule. **b** LC-MS MRM product ions are shown within each panel containing corresponding LC-MS chromatograms. MRM fragmentation of DHPAA standard matched that of the major product from wild-type (WT) DHPAAS reaction with L-DOPA. LC-MS MRM fragmentation of THP standard matched that of a major product from Phe79Tyr-Tyr80Phe-Asn192His DHPAAS reaction with L-DOPA

**Fig. 6:**
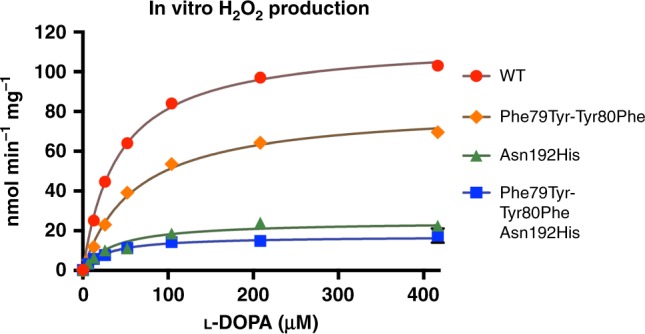
Kinetic analysis of DHPAAS H_2_O_2_ producing activities. DHPAAS produces DHPAA, CO_2_, H_2_O_2_, and NH_4_^+^ from L-DOPA, O_2_, and H_2_O. Production of H_2_O_2_ by wild-type DHPAAS and catalytic variants was monitored with a fluorometric assay. The graph was generated and analyzed with Prism 7 (*n* ≥ 8 independent measurements). The corresponding kinetic analysis is included in Table 1. Source data are provided in a Source Data file

The recombinant *B. mori* XM_004930959.2 protein produced DHPAA as the major product of L-DOPA, as indicated by the identification of negative ion *m/z* 151.10 and lack of major dopamine ions. The identification *B. mori* DHPAAS indicates that the above analysis of DHPAAS phylogenetic groups is accurate. Structural analysis supports a hypothesis that the Phe79Tyr-Tyr80Phe-Asn192His triple variant would have DDC-like activity, while Asn192His and Phe79Tyr-Ty80Phe variants would result in a mixture of DHPAAS and DDC activities. To test this hypothesis, and gain insight into a more comprehensive DHPAAS mechanism, the enzymatic activities of Phe79Tyr, Tyr80Phe, and Asn192His DHPAAS variants were evaluated (Figs 5 and 6).

Dopamine, which can be easily observed using TLC with ninhydrin staining, was identified as the major product of the Phe79Tyr-Tyr80Phe-Asn192His DHPAAS variant, supporting the above hypothesis (Supplementary Figure 4). After longer incubation times, THP was detected as the highest intensity positive ion of Phe79Tyr-Tyr80Phe-Asn192His DHPAAS reaction with L-DOPA (Fig. 5b). Comparison of DHPAAS activities via H_2_O_2_ product quantification indicates that the Asn192 residue is most important for maintaining DHPAAS activity, while Phe79 and Tyr80 residues also contribute to the DHPAAS mechanism (Fig. 6, Table 1). Characterization of aromatic products from all four DHPAAS variants was then performed in vitro.

**Table 1:** DHPAAS and DDC parameters of enzyme variants included in this study

*B. mori* DHPAAS variant	DHPAAS Km: µM L-DOPA	DHPAAS kcat: min^−1^	DHPAAS Vmax: nmol H_2_O_2_ min^−1^ mg^−1^	DDC Vmax: nmol dopamine min^−1^ mg^−1^	Estimated % DHPAAS activity
WT DHPAAS	41.7 (36.6–47.3)	6.51 (6.27–6.76)	115	5.59	95%
Phe79Tyr-Tyr80Phe	62.3 (49.5–78.5)	4.65 (4.30–5.03)	82.2	41.4	67%
Asn192His	36.5 (32.8–40.5)	1.40 (1.35–1.44)	24.7	87.6	22%
Phe79Tyr-Tyr80Phe- Asn192His	27.6 (17.1–42.8)	0.97 (0.87–1.09)	15.4	247	5.9%

**Fig. 7:**
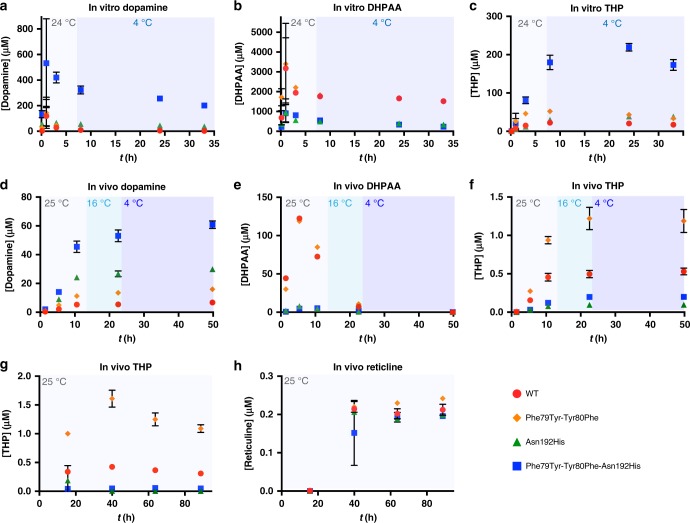
DHPAAS-mediated production of dopamine, DHPAA, THP and reticuline. **a**–**c**, In vitro DHPAAS reactions with 1.875 mM L-DOPA and 2.5 mM sodium ascorbate were incubated at 23–24 °C for 8 h, after which the reactions were moved to 4 °C. Dopamine (**a**), DHPAA (**b**), and (*R,S*)-THP (**c**) were quantified at 9 min., 1 h, 3 h, 8 h, 24 h, and 33 h after starting DHPAAS reactions. Three independent samples were measured for each in vitro point (*n* = 3). **d–f**, In vivo DHPAAS-mediated production of dopamine (**d**), DHPAA (**e**), and (*R,S*)-THP (**f**). After the addition of L-DOPA (4.92 mM, t = 0), cultures were incubated at 25 °C for initial production. 12.9 h after L-DOPA addition culture temperature was changed to 16 °C, and 22.7 h after L-DOPA addition cultures were moved to 4 °C. Products were quantified at 1.4 h, 5.4 h, 10.6 h, 22.5 h, and 49.8 h after substrate addition. Production of DHPAA was measured immediately with single measurements, as unstable DHPAA levels rapidly decreased even after freezing. Triplicate measurements were later taken for all three products after longer storage at −30 °C (*n* = 3 independent measurements). Data for in vivo triplicate measurements of DHPAA are shown in Supplementary Figure 5. **g**, **h**, Observation of an in vivo THP (**g**) to reticuline (**h**) bottleneck. M9 growth medium was supplemented with 450 µM L-DOPA for reticuline production mediated by *E. coli* containing *DHPAAS*, *6-OMT*, *CNMT*, and *4-OMT* at 25 °C. For the first time point 1 or 2 independent measurements were taken (*n* = 1 for Phe79Tyr-Tyr80Phe and Phe79Tyr-Tyr80Phe-Asn192His DHPAAS, *n* = 2 for WT and Asn192His DHPAAS). For the last three time points 2 or 4 independent measurements were taken (*n* = 2 for Phe79Tyr-Tyr80Phe and Phe79Tyr-Tyr80Phe-Asn192His DHPAAS, *n* = 4 for WT and Asn192His DHPAAS). All graphs were generated using Prism 7 with error bars representing standard deviation. Source data are provided in a Source Data file

Km, kcat, and Vmax values were calculated with Prism 7 using H_2_O_2_ production data from Fig. 6. 95% confidence intervals listed in parenthesis (*n* ≥ 8 independent measurements). DDC activity Vmax is calculated based on the first in vitro measurements of dopamine product (Fig. 7a, t = 9 min.). Estimated % DHPAAS activity is based on the comparison of these two rates.

### Direct production of THP via engineered DHPAAS

After confirming that THP could be directly produced by Phe79Tyr-Tyr80Phe-Asn192His DHPAAS, in vitro production was quantified using all four designed *B. mori* DHPAAS variants. Dopamine, DHPAA and THP were monitored using LC-MS operated in MRM mode (Fig. 7a–c). THP was highly sensitive to oxidation, as indicated by the detection of ion *m/z* 284.10, which corresponds to THP-quinone ([THP-3H]^+^ = 284.0917).

In vitro THP production improved significantly after supplementation with ascorbate to overcome product degradation via H_2_O_2_ mediated oxidation. After supplementation with 2.5 mM sodium ascorbate, conversion of L-DOPA to THP increased to 23% (219 µM) using Phe79Tyr-Tyr80Phe-Asn192His DHPAAS. This surpasses the highest bioconversion of dopamine to THP reported at 15.9%^10^. Ascorbic acid did not inhibit DHPAA production by DHPAAS indicating that DHPAA is a direct enzymatic product of L-DOPA, rather than a secondary product of dopamine oxidation by H_2_O_2_.

**Fig. 8:**
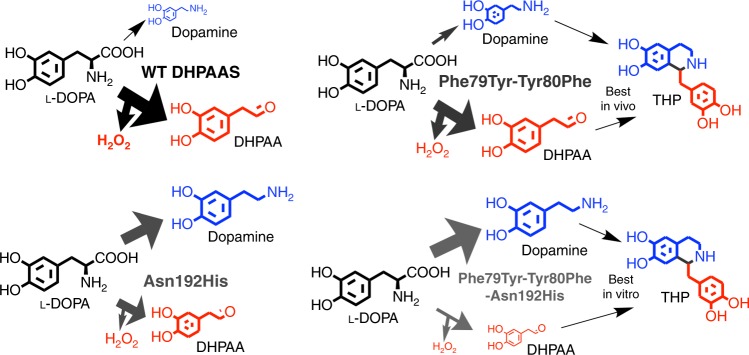
General scheme of tuning engineered DHPAAS variants for THP production. Based on the activities presented in Table 1, major products are presented as larger sized molecules relative to the scale of minor products

As predicted, overall DHPAA production was highest with wild-type DHPAAS and the Phe79Tyr-Tyr80Phe variant, and lowest with the Asn192His and Phe79Tyr-Tyr80Phe-Asn192His variants (Fig. 7a–c, Table 1). The expected opposite trend was observed for dopamine production, which was highest with the Phe79Tyr-Tyr80Phe-Asn192His variant and lowest with wild-type DHPAAS, while dopamine production by the Asn192His variant was higher than that of the Phe79Tyr-Tyr80Phe variant. Therefore, the in vitro results support the above structure-based hypothesis about the effect of Phe79, Tyr80 and Asn192 on DHPAAS mechanism switching (Table 1, Fig. 8). Additional testing was performed to further optimize DHPAAS-mediated THP bioproduction in vivo.

### Tuning DHPAA-mediated BIA bioproduction

Initial attempts at bioproduction using *E. coli* grown in lysogeny broth (LB) medium resulted in low THP titers, with higher amounts produced by the Phe79Tyr-Tyr80Phe variant, followed by that of wild-type DHPAAS. After changing the growth medium from LB to M9 minimal medium, THP production increased (Fig. 7d–f).

In vivo bioproduction of dopamine and DHPAA further supported the DHPAAS engineering model involving substitution of Phe79, Tyr80, and Asn192 (Fig. 7d–f, Supplementary Figure 5). In contrast to the in vitro results, the Phe79Tyr-Tyr80Phe variant was the best relative in vivo THP producer of this study, with titers exceeding 1 µM (0.287 mg/L). Wild-type DHPAAS produced the next highest amounts of in vivo THP, followed by Phe79Tyr-Tyr80Phe-Asn192His DHPAAS as the third-best THP producer. The Asn192His variant produced the lowest in vivo THP titers when including ascorbate. A diastereomeric mixure of (*R,S*)-THP was produced by DHPAAS in vivo as demonstrated by chiral LC-MS analysis (Supplementary Figure 6). After addition of *Coptis japonica 6-OMT*, *CNMT* and *4-OMT* genes (Fig. 1) to DHPAAS expressing *E. coli*, THP and reticuline production followed similar trends. The Asn192His and Phe79Tyr-Tyr80Phe-Asn192His DHPAAS variants resulted in higher reticuline titers than THP titers suggesting high conversion of THP to reticuline. However, reticuline titers were limited to approximately 0.2 µM using all four DHPAAS variants, which indicates that there is a bottleneck in the conversion of (*R*,*S*)-THP to reticuline under the conditions tested (Fig. 7g, h).

To improve the limited reticuline titers, additional *DHPAAS*, *DDC*, *6-OMT*, and *CNMT* genes were tested. Addition of a bacterial DDC resulted in an increase in THP titers to above 2 µM (0.57 mg/L), accompanied by a huge increase in dopamine levels. However, reticuline titers did not simply increase dependent on THP concentration, with the bottleneck appearing strongest at the 6-OMT and 4-OMT steps. Expression of DHPAAS-Phe79Tyr-Tyr80Phe-Asn192His together with wild-type DHPAAS, as well as *Coptis japonica* 6-OMT, CNMT, and 4-OMT, resulted in a moderate increase in relative THP. Yet, the bottleneck to reticuline still remained, although it appeared to be relaxed at the 6-OMT and 4-OMT steps.

**Fig. 9:**
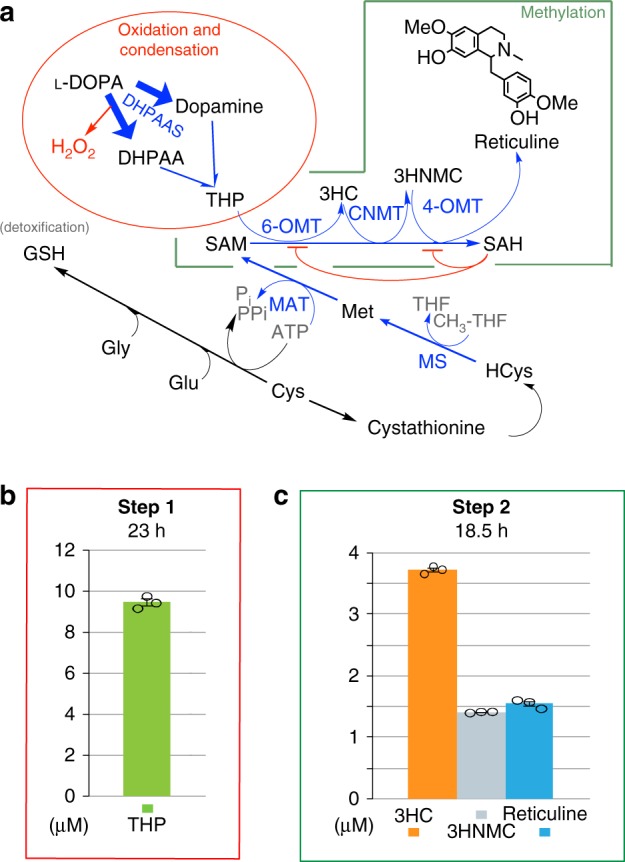
Metabolomics directed optimization of (*R*,*S*)-THP and reticuline production. **a** Monitored metabolites including homocysteine (HCys) and glutathione (GSH) are listed in black text, whereas unmeasured metabolites are listed in graey. Lower relative levels of 3-hydroxycoclaurine (3HC) and reticuline suggested that a bottleneck was strongest at 6-OMT and 4-OMT steps, which are inhibited by *S*-adenosylhomocysteine (SAH) as illustrated by the red flat-headed curves^33^. Lower utilization of *S*-adenosylmethionine (SAM) and higher accumulation of SAH accompanied stronger THP to reticuline bottlenecks. Desired pathways mediated by DHPAAS, 6-OMT, CNMT, 4-OMT, methionine synthase (MS), and methionine adenosyltransferase (MAT) are shown in blue. **b**, **c** Titers of THP, 3HC, 3-hydroxy-*N*-methylcoclaurine (3HNMC), and reticuline from 1 mM L-DOPA using a two-step cell process as described in the methods section. THP was quantified 23 h after L-DOPA adddition and three downstream BIAs were quantified 18.5 h after addition of the second bioproducer. Here, error bars define the standard error of the mean (*n* = 3 independent measurements). Source data of Fig. 9 panels **b** and **c** are provided in a Source Data file

Metabolomics analysis suggested that conversion of (*R*,*S*)-THP to reticuline may not be optimal during simultaneous conversion of L-DOPA to THP due to disruption of pathways relating to SAM cofactor recycling (Fig. 9a). Therefore a two-step cell production system was tested as described in the methods section^10,11^, in an attempt to balance the two potentially counteracting pathways. This resulted in a four- to eight-fold increase of (*R*,*S*)-THP titer to 9.45 µM (2.71 mg/L) in the first step via wild-type DHPAAS and DHPAAS-Phe79Tyr-Tyr80Phe-Asn192His. A second addition of BL21(DE3) containing 4-OMT from *C. japonica* together with 6-OMT and CNMT from *P. somniferum* was then able to relieve the bottleneck to produce 3.7 μM 3-hydroxycoclaurine (3HC), 1.4 μM 3-hydroxy-*N*-methylcoclaurine (3HNMC) and 1.5 μM reticuline after an additional 18.5 h (Fig. 9b, c). This represents a 7.5-fold increase in reticuline, as well as additional increases in 3HC and 3HNMC, demonstrating scalable titers using the DHPAAS-mediated system.

## Discussion

To scale up THP and reticuline production, improved synthetic biology design can be driven by two types of learning information: enzyme parameters and metabolomics data. Regarding learning for enzyme improvement, Phe79, Tyr80, and Asn192 were all elucidated as key residues that can tune the DHPAAS mechanism for optimized THP precursor ratios. Yet questions still remain about the exact function of these residues, including their role in O_2_ activation, which remains a somewhat mysterious activity of PLP enzymes^28^. DHPAAS and AAS utilize aromatic amino acid, O_2_ and H_2_O to produce aryl acetaldehyde, CO_2_, H_2_O_2_, and NH_4_^+^. Similarly, MAO mediates the production of aldehyde, H_2_O_2_ and NH_4_^+^ from monoamine, O_2_ and H_2_O via a flavin adenine dinucleotide (FAD) cofactor^29^. To investigate the incorporation of oxygen in the DHPAAS mechanism, DHPAAS reactions were performed in D_2_O and H_2_^18^O; however, DHPAAS activity could not be observed under these conditions. The many uncertainties about the DHPAAS and AAS mechanisms should be clarified in future studies.

DHPAAS specificity for L-DOPA was high, with no observed activity for tyrosine or phenylalanine by the Phe79Tyr-Tyr80Phe-Asn192His variant. This indicates that competitive inhibition by aromatic amino acids is not a major consideration for DHPAAS-mediated biosynthesis. However, preliminary analysis indicates possible feedback inhibition of DHPAAS by dopamine and DHPAA, and possible substrate inhibition at high concentrations of L-DOPA. These factors, together with the low measured Km of *B. mori* DHPAAS, may help to explain why L-DOPA was not consumed during in vivo bioproduction experiments. It is also possible that DHPAAS is inhibited by unknown growth medium components. Therefore, substrate concentration, substrate specificity and inhibition should be comprehensively characterized as data for enzyme learning.

During in vitro production of THP, high concentration dopamine was best, as more dopamine can quickly consume reactive DHPAA while it is being produced. Thus, Asn192His and Phe79Tyr-Tyr80Phe-Asn192His variants were observed to produce the highest THP levels in vitro. However, in vivo environments include many more cellular and chemical components that can compete with dopamine for reaction with DHPAA. Accordingly, the use of minimal medium together with higher levels of DHPAA produced by wild-type DHPAAS and the Phe79Tyr-Tyr80Phe variant resulted in better in vivo THP titers. These results highlight that the optimal dopamine to DHPAA ratio for THP production depends on the culture conditions.

The best reported THP titer of 1 mM (287 mg/L) was produced from 12.6 mM dopamine using MAO expressing cells^10^. MAO-mediated conversion of dopamine to THP was 15.9%, without reported metrics for conversion of L-DOPA to dopamine^10^. In the current study, DHPAAS expressing cells produced 9.45 μM THP (2.71 mg/L) with a yield of 1.89% from 1 mM L-DOPA. The lower titer and yield may be explained by the 12.6-fold lower substrate concentration and the additional decarboxylation step from L-DOPA. Moreover, a majority of 1 mM L-DOPA remained unconsumed in the culture medium, suggesting low substrate permeability and possibly higher intracellular yields.

L-DOPA utilization by DHPAAS is much better in vitro, with 23% conversion of L-DOPA to 219 µM THP. In the MAO-mediated system, a THP titer of 99.2 µM resulted in the highest reticuline titers^11^. Therefore, in vitro results suggest that DHPAAS-mediated THP production is capable of reaching optimal levels for downstream BIA production. However, DHPAAS-mediated in vivo production needs to be improved to reach the 1 mM THP benchmark and computational yields greater than 50%.

The DHPAAS-mediated pathway also requires improvements in downstream BIA production. After the establishment of proof-of-principle bioproducers, it usually requires more than five years of optimization to reach industrial demands^30^. Indeed, the complete pathway is now assembled into a single *E. coli* cell to produce high titer reticuline from glucose^31^, 10 years after the development of the first MAO-mediated reticuline producing system^32^. To better match this benchmark, a complete DHPAAS-mediated pathway from glucose to (*S*)-reticuline is now under development. Metabolomics-directed learning data should include flux from toxic intermediates DHPAA^20^ and THP^10^ towards less-toxic reticuline. Addition of NCS can increase conversion of DHPAA to selectively produce (*S*)-THP, leading to better substrate conversion by SAM dependent BIA methyltranferases^31,33^. A related OMT bottleneck with impaired SAM cofactor recycling was reported for vanillin biosynthesis in *E. coli* grown on M9 minimal medium^34^. This supports balancing of SAM recycling pathways as another strategy to improve reticuline production (Fig. 9). Quenching of H_2_O_2_ by antioxidant enzymes including catalase II (katE) should also be pursued to increase BIA titers.

Additional M-path analysis was performed to search alternative bioproduction pathways that might be improved using DHPAAS or AAS, linking the learning process of enzyme database improvement to new cycles of pathway design. For example, M-path identified EC 4.2.1.107–109 as enzymes to mediate biosynthesis of 2’-norberbamunine^35^, homovanillic acid, imidazole-4-acetaldehyde, and 4-amino-phenylacetaldehyde. In conclusion, this study demonstrates that functional enzyme engineering can be effectively applied to high value bioproduction pathways. Most importantly, further development of the DHPAAS-mediated pathway is required to improve downstream BIA production in future studies.

## Methods

### M-path prediction of enzymes and pathways

Enzyme searches were performed using the M-path web-based version according to the methods of Araki et al.^14^. M-path scores are calculated as a Tanimoto co-efficient. The 2016 version of the M-path database was updated using the most recent substrate, product, and enzyme information from KEGG and BRENDA. Curated mode was used to search for enzymes to mediate tyrosine (PubChem CID: 6057) to THP (CID: 18519), tyrosine to 4-HPAA (CID: 440113), L-DOPA (CID: 6047) to THP, L-DOPA to DHPAA (CID: 119219), tyrosine to 2′-norberbamunine (CID: 441063), histidine (CID: 6274) to imidazole-4-acetaldehyde (CID: 150841), and 4-aminophenylalanine (CID: 151001) to 4-aminophenylacetaldehyde (CID: 20440863). For conversion of tyrosine to homovanillic acid (CID: 1738), M-path search was performed in original mode.

### Computational prediction of enzyme-mediated THP production

Mathematical models were constructed for the nonsymmetrical (DDC-MAO) and symmetrical (DDC-DHPAAS) pathways. For the nonsymmetrical pathway, since MAO recognizes a variety of amines, competitive inhibition was introduced into the MAO reaction velocity V_MAO_^12,13^. Models were constructed with and without product feedback inhibition factored into the reaction velocities of MAO (V_MAO_), DDC (V_DDC_) and DHPAAS (V_DHPAAS_). Drain of DHPAA was later included in some models. Possible ranges of parameter values were surveyed and collected from literature and BRENDA^15^. In order to predict the performance of each pathway, parameter values were randomly generated within the set ranges, and Monte–Carlo simulation was carried out according to Supplementary Methods. The iteration number was 10,000, and simulation time was 0–50 h. L-DOPA was fed as a constant term based on randomly generated parameters. When the maximum amount of 100 mM L-DOPA was reached, substrate feeding to the system was stopped. A homemade program was implemented in Python 3.0 using scipy.integrate.odeint as the solver for numerical simulations. More detailed computational methods are described in Supplementary Methods, Supplementary Figures 2–3, and Supplementary Table 2.

### Homology modeling and phylogenetic analysis

Dimeric homology models of *B. mori* DHPAAS and *P. somniferum* TyDC1 were generated with MODELLER^22^ operated within Chimera^23^. Crystal structures of *D. melanogaster* DDC (PDB ID: 3K40)^25^ and histidine decarboxylase (4E1O)^26^ were used as templates for *B. mori* DHPAAS modeling. The structure of *Sus Scrofa* DDC in complex with carbidopa (PDB ID: 1JS3)^24^ was used as a template for TyDC1. Binding of PLP covalently linked to aromatic amino acid substrate, and structure refinement were performed with Molecular Operating Environment (MOE). Completed structures were analyzed in PyMOL.

For phylogenetic analysis, insect AAAD and DHPAAS sequences were collected from the protein BLAST non-redundant database after searching from insect sequences NP_476592.1, NP_724162.1, XP_319838.3, EDS39158.1, EAT37246.1, and EAT37247.1. Duplicate sequences, sequences containing X as an amino acid, and sequences over 700 amino acids in length were all removed. The resulting sequences were aligned and a phylogenetic tree was generated using a split value of 0.12. After removing partial sequences, clusters were identified from a sequence similarity table generated by MOE.

### Preparation of recombinant *B. mori* DHPAAS

The full length *B. mori DHPAAS* native sequence was synthesized by GeneArt (Invitrogen) and cloned into the pE-SUMO vector with kanamycin resistance (LifeSensors Inc.) via BsaI restriction sites. Mutations were generated using overlapping PCR with primers shown in Supplementary Table 4. DHPAAS expression vectors were transformed into BL21(DE3) maintained in LB supplemented with 50 µg/mL kanamycin, or BL21(DE3)pLysS maintained in LB supplemented with 50 µg/mL kanamycin and 34 µg/mL chloramphenicol. Expression of recombinant DHPAAS was induced by the addition of 0.2–0.45 mM IPTG to *E. coli* grown aerobically in LB medium. After induction, culture temperature was lowered to 14–16 °C. After overnight incubation, cells were pelleted by centrifugation, resuspended in phosphate-buffered saline (PBS) and lysed by sonication while being cooled over ice. After centrifugation, clarified lysates were loaded onto HiTrap TALON or HisTrap HP columns (GE Life Sciences). After loading, columns were washed with PBS and 10–20 mM imidazole. Recombinant DHPAAS was eluted with 450–1000 mM imidazole. Buffer was changed to PBS supplemented with PLP using Amicon Ultra-15 centrifugal filters (Millipore).

### TLC analysis of DHPAAS reactions

TLC was performed on aluminum plates coated with silica gel 60 F254 (Merck Millipore). A mixture of 1-butanol, acetic acid and H_2_O at a ratio of 7:2:1 was used as the mobile phase. DHPAAS reaction components were analyzed under UV light before ninhydrin staining.

### Mass analysis of DHPAAS substrates and products

Substrate and products were identified with Shimadzu LCMS-8050 and LCMS-8060 ESI triple quadrupole mass spectrometers. Quantitative analysis was performed using the Shimadzu LCMS systems operated in multiple reaction monitoring (MRM) mode with the following parameters: ionization, ESI; DL temp., 250 °C; block heater temp., 400 °C; interface temp., 300 °C; nebulizing gas flow, 3.0 L/min; drying gas flow, 10.0 L/min; heating gas flow, 10.0 L/min. The ion source was connected to a Shimadzu Nexera X2 UHPLC system^36^. Five concentrations of fresh L-DOPA (TCI), dopamine (TCI), DHPAA (Santa Cruz Biotechnology), THP hydrobromide (Sigma) and reticuline were analyzed to generate quantitative standard curves. Quantifier MRM transitions of 198.10 > 152.10(+), 154.10 > 91.05(+), 151.30 > 123.15(−), and 288.05 > 164.15(+) were used for L-DOPA, dopamine, DHPAA and THP, respectively. Qualifier MRM transitions of 154.10 > 137.05(+), 151.30 > 122.10(−), and 288.05 > 123.15( + ) were used for dopamine, DHPAA and THP, respectively. MRM transitions of 330.10 > 192.00( + ), 330.10 > 137.15( + ) and 330.10 > 177.20(+) were used for reticuline. Separation was performed at 0.25 mL/min on a Discovery HS F5-3 column (3 µm, 2.1 × 150 mm, Sigma–Aldrich) heated to 40 °C, with a mobile phase gradient of 0.1% formic acid in water to 0.1% formic acid in acetonitrile. Chiral analysis of (*R,S*)-THP was performed using the Shimadzu LCMS-8050 system operated in MRM mode, together with an Astec CYCLOBOND I 2000 column (5 µm, 2.1 × 150 mm, Sigma–Aldrich) heated to 35 °C and a mobile phase gradient of 90% acetonitrile to 50 mM NH_4_OAc (pH 4.5) flowing at 0.3 mL/min.

### In vitro quantification of DHPAAS reaction components

For quantification of aromatic DHPAAS substrates and products, soluble DHPAAS (2–3 µg) in PBS was mixed with an aqueous solution of L-DOPA to a final volume of 40 µL. A final concentration of 1.875 mM L-DOPA was used together with 2.5 mM sodium ascorbate (Wako). Reactions were started at room temperature (23–24 °C) and transferred to 4 °C after 8 h. At various time points, 2 µL of each reaction was diluted into 98 uL methanol supplemented with ascorbate and camphor sulfonic acid standard. Dilutions were performed in triplicate. DHPAAS reaction dilutions were immediately stored at −30 °C until triplicate LC-MS analysis. In vitro data were analyzed with Prism 7.

H_2_O_2_ production was analyzed in 96 well plates using a fluorometric hydrogen peroxide assay kit (Sigma). 0.6–0.8 µg soluble DHPAAS in PBS (20 µL) was mixed with varying concentrations of L-DOPA (10 µL), followed by the addition of 30 µL peroxidase enzyme and fluorescent substrate mix (Sigma). Fluorescence was detected with a SpectraMax Paradigm microplate reader (Molecular Devices). Kinetic data included eight independent conditions for wild-type DHPAAS (one outlier), Asn192His variant (two outliers), and Phe79Tyr-Tyr80Phe variant, while 15 independent conditions were used for the Phe79Tyr-Tyr80Phe-Asn192His variant. Kinetic data was analyzed using the kcat function of Prism 7 with outlier elimination.

### In vivo DHPAAS-mediated THP bioproduction

*DHPAAS* sequences were PCR amplified with primers containing NcoI and XhoI restriction sites (Supplementary Table 4) for cloning into pTrcHis2B. The resulting untagged expression vectors were then transformed into BL21(DE3)pLysS. For bioproduction, cells were grown in M9 medium supplemented with 2% glucose, 15.6 mM sodium ascorbate, 100 µg/mL ampicillin, and 34 µg/mL chloramphenicol. Cultures containing 3.5 mL medium were grown at 37 °C with shaking. When culture OD_600_ reached 0.2–0.4, DHPAAS expression was induced with 0.97 mM IPTG, and the growth temperature was lowered to 25 °C. At 1 h, 13 min post-induction, 3.4 mg L-DOPA (0.97 mg/mL, 4.92 mM) was then added to each culture, followed by addition of PLP to a final concentration of 4.86 µM. Culture temperature was decreased to 16 °C 12.9 h. after the addition of L-DOPA. Culture samples (300–500 µL) for four time points were filtered through Amicon Ultra 0.5 mL centrifugal filters (Millipore) with a molecular weight cut-off of 3000 Da. Approximately 4–5 mg ascorbate was added to each culture 22.7 h. after substrate addition, and cultures were moved to 4 °C. Finally, 49.8 h. after substrate addition, cultures were centrifuged at 4500 × *g*, followed by collection and analysis of supernatants. Culture samples were diluted in methanol with camphor sulfonic acid and ascorbate for quantification of dopamine, DHPAA and THP. Individual dilutions were analyzed three times for each condition, and in vivo figures with error bars were generated using Prism 7.

### In vivo DHPAAS-mediated reticuline bioproduction

pTrcHis2B containing *DHPAAS* was transformed, together with a pACYC184 derived vector containing *C. japonica 4-OMT*, *CNMT*, and *6-OMT*, into BL21(DE3). Resulting *E. coli* reticuline bioproducers were selected with 100 µg/mL ampicillin and 34 µg/mL chloramphenicol. Reticuline production was tested in M9 minimal medium supplemented with 2% glucose. Cells were grown to an OD_600_ of 0.2–0.3 before the addition of 0.5 mM IPTG, 450 µM L-DOPA, and 4.54 mM sodium ascorbate. 17.2 h. after substrate addition PLP was added to 54.8 µM along with a second addition of an additional 444 µM ascorbate. SAM was added 23.5 h after substrate addition to a final concentration of 9.7 µM. Reticuline was produced at 25 °C with shaking. Filtered culture medium was diluted in acetonitrile with camphor sulfonic acid and ascorbate for quantification of dopamine, DHPAA, THP, and reticuline. Single cultures of Phe79Tyr-Tyr80Phe and Phe79Tyr-Tyr80Phe-Asn192His DHPAAS containing *E. coli* were tested. Duplicate cultures of wild-type and Asn192His DHPAAS containing *E. coli* were tested. Each dilution was measured in duplicate, resulting in duplicate measurements for Phe79Tyr-Tyr80Phe and Phe79Tyr-Tyr80Phe-Asn192His DHPAAS-mediated reticuline production, and quadruplicate measurements for wild-type and Asn192His DHPAAS-mediated reticuline production. In vivo figures with error bars were generated using Prism 7.

For optimization of reticuline titers, 3HC and 3HNMC (Toronto Research Chemicals) were analyzed together with DHPAA, THP, reticuline, and 115 central metabolites on the Shimadzu LCMS-8050^36^.

### Two-step cell production of BIA

BL21(DE3) was transformed with wild-type *DHPAAS* in pTrcHis2B, *DHPAAS-Phe79Tyr-Tyr80Phe-Asn192His* in pE-SUMO, and *C. japonica 4-OMT*, *CNMT* and *6-OMT* in pACYC184. For the first step of THP production, this three plasmid system was initially grown at 37 °C in TB with no glycerol supplemented with 1.5% glucose, 100 µg/mL ampicillin, and 50 µg/mL kanamycin. After reaching OD_600_ 0.38, IPTG was added to a final concentration of 0.5 mM IPTG. 1.5 h after induction, the temperature was lowered to 25 °C. At 5.5 h post-induction, cells were collected by centrifugation at 4000 g, stored overnight at −80 °C, and the pellet from ~43 mL culture was resuspended in M9 with reduced calcium, 0.2% Triton X-100, 1.5% glucose, 10 μM PLP, 10 mM sodium ascorbate, and 1 mM L-DOPA, to a final volume of 6.5 mL. After mixing, cultures were kept at 24–25 °C for 1.5 h and then centrifuged at 5000 × *g* to assist condensation of DHPAA with dopamine in the supernatant. Twenty-five hours after substrate addition, the static culture was centrifuged again at 5000 × *g* and the THP containing supernatant was used for the next downstream BIA production step.

For the second step of increased downstream BIA production, pET23a containing *4-OMT* from *C. japonica* together with *6-OMT* and *CNMT* from *P. somniferum* was transformed into BL21(DE3). This second BIA producer was also initially grown at 37 °C in TB with no glycerol supplemented with 1.5% Glucose and 100 µg/mL ampicillin. After reaching OD_600_ 0.78, IPTG was added to a final concentration of 0.5 mM. 1.5 h after induction, the temperature was lowered to 25 °C. 5.5 h post-induction, cells were collected by centrifugation at 4000 × *g*, stored for 2 nights at −80 °C, and the pellet from ~46 mL culture was resuspended in the supernatant from the first step. Downstream BIA production was then tested at 25 °C with shaking. Diluted samples of culture medium were analyzed with three independent measurements for each condition.

### Reporting summary

Further information on experimental design is available in the Nature Research Reporting Summary linked to this article.

## Supplementary information

Supplementary Information

Peer Review

Reporting Summary

Source Data

## Data Availability

Data supporting the findings of this work are available within the paper and its Supplementary Information files. A reporting summary for this Article is available as a Supplementary Information file. The datasets generated and analyzed during the current study are available from the corresponding author upon reasonable request. The source data underlying Figs. 6, 7, 9b, and 9c, as well as Supplementary Figure 5 are provided as a Source Data file.

## Code availability

The computer code used for metabolomic modeling [https://github.com/yukuriya3/THP_pro_pathway_select] and numerical simulation [https://github.com/yukuriya3/THP_pro_pathway_select] are available on GitHub.
