# Author Correction: Mechanism-based tuning of insect 3,4-dihydroxyphenylacetaldehyde synthase for synthetic bioproduction of benzylisoquinoline alkaloids

**DOI:** 10.1038/s41467-019-10312-y

**Published:** 2019-05-22

**Authors:** Christopher J. Vavricka, Takanobu Yoshida, Yuki Kuriya, Shunsuke Takahashi, Teppei Ogawa, Fumie Ono, Kazuko Agari, Hiromasa Kiyota, Jianyong Li, Jun Ishii, Kenji Tsuge, Hiromichi Minami, Michihiro Araki, Tomohisa Hasunuma, Akihiko Kondo

**Affiliations:** 1 Graduate School of Science, Technology and Innovation, Kobe University, 1-1 Rokkodai-cho, Nada-ku, Kobe, 657-8501 Japan; 2 Mitsui Knowledge Industry Co., Ltd. (MKI), 2-3-33 Nakanoshima, Kita-ku, Osaka, 530-0005 Japan; 3 Graduate School of Medicine, Kyoto University, Yoshida-Konoe-cho, Sakyo-ku, Kyoto, 606-8501 Japan; 4 Graduate School of Environmental and Life Science, Okayama University, 1-1-1, Tsushima-Naka, Kita-ku, Okayama, 700-8530 Japan; 5 Department of Biochemistry, Virginia Polytechnic and State University, 111 Engel Hall, Mail Code: 0308, Blacksburg, VA 24061 USA; 6 Research Institute for Bioresources and Biotechnology, Ishikawa Prefectural University, 1-308, Suematsu, Nonoichi-shi, Ishikawa-ken, 921-8836 Japan; 7 Engineering Biology Research Center, Kobe University, 1-1 Rokkodai-cho, Nada-ku, Kobe, 657-8501 Japan; 8 Department of Chemical Science and Engineering, Graduate School of Engineering, 1-1 Rokkodai-cho, Nada-ku, Kobe, 657-8501 Japan

**Keywords:** Enzyme mechanisms, Metabolic engineering, Applied microbiology, Synthetic biology

Correction to: *Nature Communications* 10.1038/s41467-019-09610-2, published online 01 May 2019.

In the original version of this Article, the abbreviation of 3,4-dihydroxyphenylacetaldehyde synthase presented in the first paragraph of the Discussion section was given incorrectly as DYPAA. The correct abbreviation for this enzyme is DHPAAS. This error has been corrected in both the PDF and HTML versions of the Article.

