# Supplementary material for: Mechanism-based tuning of insect 3,4-dihydroxyphenylacetaldehyde synthase for synthetic bioproduction of benzylisoquinoline alkaloids
Source: Nat Commun. 2019 May 1;10:2015. doi: 10.1038/s41467-019-09610-2 (PMC6494836; doi:10.1038/s41467-019-09610-2)
Supplement: Supplementary file 1 — Supplementary Information [file 41467_2019_9610_MOESM1_ESM.docx]

**Mechanism-based tuning of insect 3,4-dihydroxyphenylacetaldehyde synthase for synthetic bioproduction of benzylisoquinoline alkaloids**

Vavricka *et al.*

**Supplementary Methods**

**Kinetic models of tetrahydropapaveroline production**

Simple mathematical models were constructed for nonsymmetrical and symmetrical pathways to tetrahydropapaveroline (THP) from L-3,4-dihydroxyphenylalanine (L-DOPA). Competitive inhibition by amines was factored into the monoamine oxidase (MAO) catalyzed reaction velocity, with the inhibition term described as lamped parameter “Inh” (Eq. **7**). MAO models were later included with product feedback inhibition. Two models were constructed for the symmetrical pathway, one with no feedback and one with product feedback inhibition factored into the L-DOPA decarboxylase (DDC) and 3,4-dihydroxyphenylacetaldehyde synthase (DHPAAS) reaction velocities (V_DDC_ and V_DHPAAS_). L-DOPA substrate was fed as a constant term. In vivo experiments indicated that 3,4-dihydroxyphenylacetaldehyde (DHPAA) is readily depleted by competing reactions, and this was later included in additional models as V_drain_. Mass balance equations are shown below.

Nonsymmetrical pathway model mass balance equations

$\frac{d[L-Dopa]}{dt}=v_{fed}-v_{DDC}$ (**1**)

$\frac{d[\mathrm{Dopamine}]}{dt}=v_{DDC}-v_{MAO}-v_{spon}$ (**2**)

$\frac{d[\mathrm{DHPAA}]}{dt}=v_{MAO}-v_{spon}-v_{drain}$ (**3**)

$\frac{d[THP]}{dt}=v_{spon}-v_{cons}$ (**4**)

Nonsymmetrical rate equations

$v_{fed}=k_{fed}$ (**5**)

Rates without feedback inhibition

$v_{DDC}=\frac{V_{max\_MAO}\left[ L-Dopa \right]}{K_{m\_L-Dopa}+\left[ L-Dopa \right]}$ (**6**)

$v_{MAO}=\frac{V_{max\_MAO}\left[ \mathrm{Dopamine} \right]\left[ O_{2} \right]}{\left\{ K_{m\_Dopamine}\left( 1+\mathrm{Inh} \right)+\left[ \mathrm{Dopamine} \right] \right\}\left( K_{m\_O2}+\left[ O_{2} \right] \right)}$ (**7**)

Rates with feedback inhibition

$v_{DDC}=\frac{V_{max\_DDC}\left[ L-Dopa \right]}{\left\{ K_{m\_L-Dopa}\left( 1+\left[ \mathrm{Dopamine} \right]/{K_{i\_Dopamine}} \right)+\left[ L-Dopa \right] \right\}}$ (**8**)

$v_{MAO}=\frac{V_{max\_MAO}\left[ \mathrm{Dopamine} \right]\left[ O_{2} \right]}{\left\{ K_{m\_Dopamine}\left( 1+{Inh+\left[ \mathrm{DHPAA} \right]}/{K_{i\_DHPAA}} \right)+\left[ \mathrm{Dopamine} \right] \right\}\left( K_{m\_O_{2}}+\left[ O_{2} \right] \right)}$ (**9**)

$v_{spon}=k_{spon}\left[ \mathrm{Dopamine} \right]\left[ \mathrm{DHPAA} \right]$ (**10**)

$v_{cons}=k_{cons}\left[ \mathrm{THP} \right]$ (**11**)

$v_{drain}=k_{drain}\left[ \mathrm{DHPAA} \right]$ (**12**)

Symmetrical pathway model mass balance equations

$\frac{d[L-Dopa]}{dt}=v_{fed}-v_{DDC}-v_{DHPAAS}$ (**13**)

$\frac{d[Dopamine]}{dt}=v_{DDC}-v_{spon}$ (**14**)

$\frac{d[DHPAA]}{dt}=v_{DHPAAS}-v_{spon}-v_{drain}$ (**15**)

$\frac{d[THP]}{dt}=v_{spon}-v_{cons}$ (**16**)

Symmetrical rate equations

$v_{fed}=k_{fed}$ (**17**)

Rates without feedback inhibition

$v_{DDC}=\frac{V_{max\_DDC}\left[ L-Dopa \right]}{K_{m\_L-Dopa}+\left[ L-Dopa \right]}$ (**18**)

$v_{DHPAAS}=\frac{V_{max\_DHPAAS}\left[ L-Dopa \right]\left[ O_{2} \right]}{\left( K_{m\_L-Dopp\_2}+\left[ L-Dopa \right] \right)\left( K_{m\_O_{2}}+\left[ O_{2} \right] \right)}$ (**19**)

Rates with feedback inhibition

$v_{DDC}=\frac{V_{max\_DDC}\left[ L-Dopa \right]}{\left\{ K_{m\_L-Dopa}\left( 1+\left[ \mathrm{Dopamine} \right]/{K_{i\_Dopamine}} \right)+\left[ L-Dopa \right] \right\}}$ (**20**)

$v_{DHPAAS}=\frac{V_{max\_DHPAAS}\left[ L-Dopa \right]\left[ O_{2} \right]}{\left\{ K_{m\_L-Dopa\_2}\left( 1+\left[ \mathrm{DHPAA} \right]/{K_{i\_DHPAA}} \right)+\left[ L-Dopa \right] \right\}\left( K_{m\_O_{2}}+\left[ O_{2} \right] \right)}$ (**21**)

$v_{spon}=k_{spon}\left[ \mathrm{Dopamine} \right]\left[ \mathrm{DHPAA} \right]$ (**22**)

$v_{cons}=k_{cons}\left[ \mathrm{THP} \right]$ (**23**)

$v_{drain}=k_{drain}\left[ \mathrm{DHPAA} \right]$ (**24**)

**Monte Carlo simulation**

The mathematical models involve inhibition by products and competing substrates, consumption of aldehyde by side reactions (drain), and parameter uncertainties. Therefore, we performed Monte Carlo simulation to predict THP production yield from L-DOPA. The possible range of parameter values ​​in the model was surveyed and collected from literature and the BRENDA database^1^ (Supplementary Table 2). In the Monte Carlo simulation, unknown parameter values ​​for mathematical models were generated as uniform random numbers within each preset parameter range. Numerical simulations of the mathematical models were carried out using the respective parameter sets, with 10,000 iterations for each model. Histograms and box plots for THP yields were generated and results were not normally distributed. Though minimum and maximum values were similar for each model, modal values were not considered as the best representative values based on the distribution shapes of each model. Therefore, median values were taken as a better index for the evaluation of each model.

**Supplementary Figure 1. Explored pathways from L-DOPA to THP.** (**A**) Nonsymmetrical pathway mediated by DDC and MAO with inhibition. (**B**) Symmetrical pathway mediated by DDC and DHPAAS without feedback regulation. (**C**) DDC and DHPAAS pathway with feedback regulation. Abbreviations and terms are as follows: V, reaction rate; fed, substrate feeding; drain, DHPAA depletion by side reactions; spon, non-enzymatic spontaneous reaction; cons, consumption. Arrows represent reactions, while the red flat-ended curves and blue line represent inhibition.

$$v_{0}=k_{0}$$

$$v_{1}=\frac{V_{\max\_1}\left[ A \right]}{K_{mA}+\left[ A \right]}$$

$$v_{2}=\frac{V_{\max\_2}\left[ B \right]\left[ O_{2} \right]}{\left\{ K_{mB}\left( 1+Inh \right)+\left[ B \right] \right\}\left( K_{mO_{2}}+\left[ O_{2} \right] \right)}$$

$$v_{3}=k_{3}\left[ B \right]\left[ C \right], v_{4}=k_{4}\left[ D \right]$$

$$\frac{d\left[ A \right]}{dt}=v_{0}-v_{1}$$

$$\frac{d\left[ B \right]}{dt}=v_{1}-v_{2}-v_{3}$$

$$\frac{d\left[ C \right]}{dt}=v_{2}-v_{3}$$

$$\frac{d\left[ D \right]}{dt}=v_{3}-v_{4}$$

$$k_{1, j}=k_{0}=\left[ a, b \right]$$

$$k_{2, j}=V_{max1}=\left[ c, d \right]$$

$$k_{3, j}=K_{mA}=\left[ e, f \right]$$

$$\vdots$$

$$k_{n, j}=k_{4}=\left[ q, r \right]$$


**Supplementary Figure 2. Workflow for prediction of metabolic pathway performance.** (**A**) Models are constructed based on reported data together with assumptions about pathways, reactions and regulations. (**B**) Parameter value ranges are preset for Monte Carlo simulation. (**C**) Simulations are iterated with parameters randomly generated within preset ranges. (**D**) Simulation results are evaluated based on an evaluation index of THP yield. (**E**) Evaluation results are displayed as histograms and boxplots.

**Supplementary Figure 3. Histograms of L-DOPA to THP conversion efficiency based on Monte Carlo simulations.** (**A**) Results for the nonsymmetrical pathway (DDC + MAO) with inhibition. (**B**) Results for the symmetrical pathway (DDC + DHPAAS) without product feedback inhibition. (**C**) Results for the symmetrical pathway (DDC + DHPAAS) with product feedback inhibition. All statistics were derived from n = 10,000 independent Monte Carlo simulations.

**Supplementary Figure 4. TLC analysis of Phe79Tyr-Tyr80Phe-Asn192His DHPAAS conversion of L-DOPA.**

**
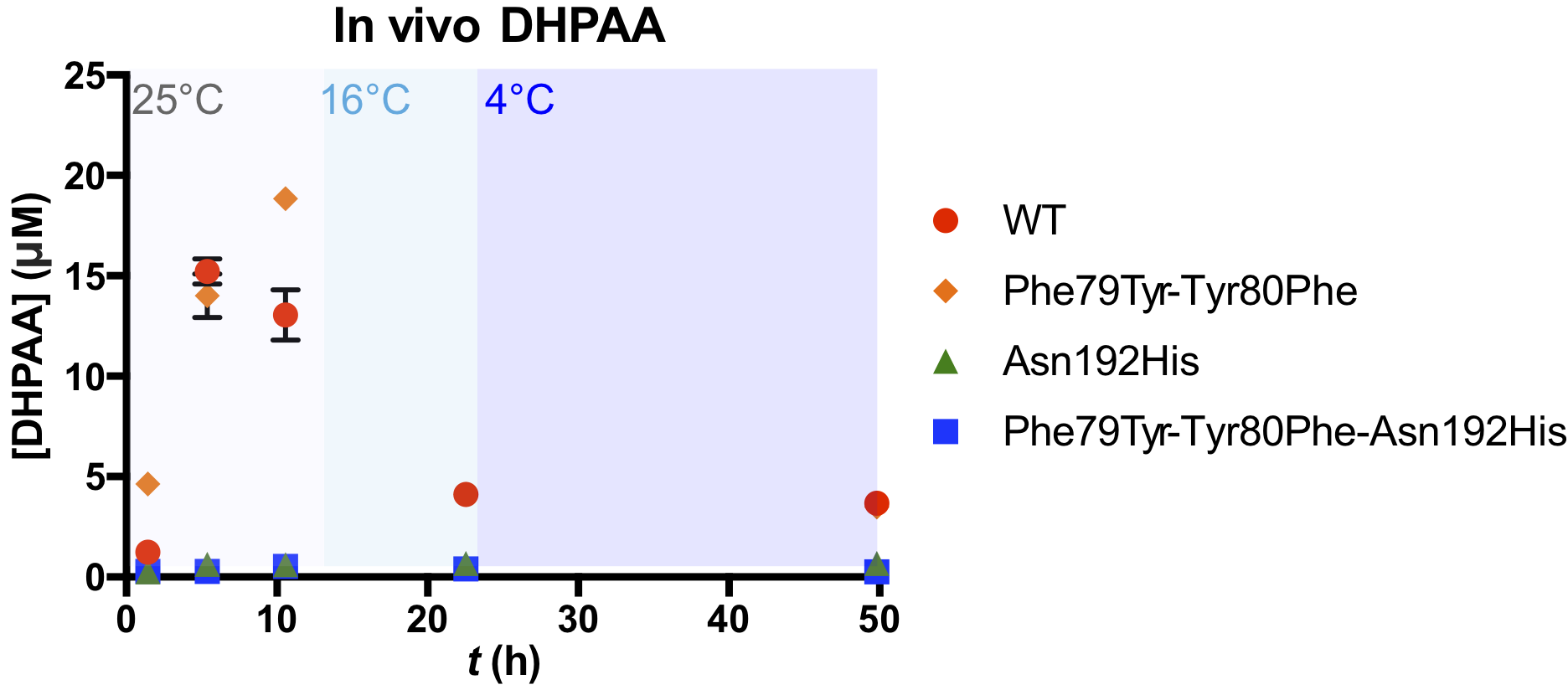
**

**Supplementary Figure 5. Triplicate analysis of in vivo DHPAA.** Quantification of DHPAAS was performed after storage at -30 °C. The graph was generated with Prism 7 with error bars representing standard deviation (n=3 independent measurements). Source data are provided in a Source Data file.

**Supplementary Figure 6**. **(*R,S*)-THP chiral LC-MS analysis.** An Astec CYCLOBOND I 2000 column with underivatized β-cyclodextrin bound to silica gel was used for the chiral analysis. The diastereomeric ratio of (*R,S*)-THP produced by DHPAAS in vivo was similar to that of chemically synthesized THP hydrobromide. The ratio of peak areas for (*R,S*)-THP are listed.

**Supplementary Table 1. M-path identification of AAS enzymes**

| M-path Reaction | PAAS Score | 4-HPAAS Score | DHPAAS Score |
| --- | --- | --- | --- |
| Tyr -> 4-HPAA | 0.621 | 1.0 | 0.763 |
| L-DOPA -> DHPAA | 0.486 | 0.763 | 1.0 |

Abbreviations are as follows: AAS, aromatic aldehyde synthase; PAAS, phenylacetaldehyde synthase; 4-HPAA, 4-hydroxyphenylacetaldehyde; 4-HPAAS, 4-HPAA synthase.

**Supplementary Table 2. Ranges of parameters in constructed models**

| Nonsymmetrical pathway model | | | | Symmetrical pathway model | | | |
| --- | --- | --- | --- | --- | --- | --- | --- |
| Name | Range | Unit | Source | Name | Range | Unit | Source |
| $k_{fed}$ | 0-10 | h^-1^ | ** | $k_{fed}$ | 0-10 | h^-1^ | ** |
| $V_{max\_DDC}$ | 0-10000 | mM h^-1^ | ** | $V_{max\_DDC}$ | 0-10000 | mM h^-1^ | ** |
| $K_{m\_L-Dopa}$ | 0-10 | mM | * | $K_{m\_L-Dopa}$ | 0-10 | mM | * |
| $V_{max\_MAO}$ | 0-10000 | mM h^-1^ | ** | $K_{i\_Dopamine}$ | 0-10 | mM | ** |
| $K_{m\_Dopamine}$ | 0-10 | mM | * | $V_{max\_DHPAAS}$ | 0-10000 | mM h^-1^ | ** |
| $K_{m\_O_{2}}$ | 0-1.0 | mM | * | $K_{m\_L-Dopa\_2}$ | 0-10 | mM | * |
| $\left[ O_{2} \right]$ | 0-0.240 | mM | *** | $K_{m\_O_{2}}$ | 0-1.0 | mM | * |
| $\mathrm{Inh}$ | 0-100 | - | ** | $\left[ O_{2} \right]$ | 0-0.240 | mM | *** |
| $k_{spon}$ | 0-1.0 | mM^-1^h^-1^ | ** | $K_{i\_DHPAA}$ | 0-10 | mM | ** |
| $k_{cons}$ | 0-1.0 | h^-1^ | ** | $k_{spon}$ | 0-1.0 | mM^-1^h^-1^ | ** |
| $K_{i\_Dopamine}$ | 0-10 | mM | ** | $k_{cons}$ | 0-1.0 | h^-1^ | ** |
| $k_{drain}$ | 0-1.0 | h^-1^ | ** | $k_{drain}$ | 0-1.0 | h^-1^ | ** |

*Based on BRENDA database. **Arbitrary values. ***Based on oxygen saturation concentration in aqueous solutions at 298.15 K ^2^.

**Supplementary Table 3. Comparison of key residues in DHPAAS and DDC**

| Group | Protein Name | Sequence Name | 80-loop | 192-region |
| --- | --- | --- | --- | --- |
| Formicidae | *C. floridanus* DHPAAS | XP_011261389.1 | HA**FY**PT | QS**N**SSV |
| *Apis* | *A. mellifera* DHPAAS | XP_006563197.1 | YA**FY**PT | QS**N**SSV |
| Lepidoptera | *B. mori* DHPAAS | XM_004930959.2 | HA**FY**PS | QC**N**SSV |
| Mosquito | *A. aegypti* DHPAAS | XP_001661057.2 | HA**FY**PS | QS**N**SAV |
| *Drosophila* | *D. melanogaster* DHPAAS | NP_724162.1 | NA**FY**PS | QS**N**SCI |
| *Drosophila* | *D. melanogaster* DHPAAS | NP_476592.1 | HA**YY**PT | QS**N**SCI |
| Formicidae | *C. floridanus* DDC | EAG_03314 | HA**YF**PT | QA**H**SSV |
| *Apis* | *A. mellifera* DDC | XP_394115.2 | HA**YF**PT | QA**H**SSV |
| Lepidoptera | *B. mori* DDC | NP_001037174.1 | HA**YF**PT | QA**H**SSV |
| Mosquito | *A. aegypti* DDC | AAC31639.1 | HA**YF**PT | QS**H**SSV |
| *Drosophila* | *D. melanogaster* DDC | AAF53764.3 | HA**YF**PT | QA**H**SSV |

**Supplementary Table 4. Primers for cloning and mutagenesis of DHPAAS**

| Primer | Sequence |
| --- | --- |
| *DHPAAS-NcoI-F* | ATATCCATGGACGCGAACCAGTT |
| *DHPAAS-XhoI-R* | GTATCTCGAGTTATTACTTAGATTTTTCGCATAG |
| *DHPAAS-C574A-F* | CTTATACATCCGACCAATGTAACTCGTCAGTAGAAAAAGC |
| *DHPAAS-C574A-R* | GCTTTTTCTACTGACGAGTTACATTGGTCGGATGTATAAG |
| *DHPAAS-A236T-T239A-F* | GCAGTTCCATGCATTCTATCCTTCTGGCTCCTC |
| *DHPAAS-A236T-T239A-R* | GAGGAGCCAGAAGGATAGAATGCATGGAACTGC |

**Supplementary References**

1. Placzek, S., Schomburg, I., Chang, A., Jeske, L., Ulbrich, M., Tillack, J., & Schomburg, D. BRENDA in 2017: new perspectives and new tools in BRENDA. *Nucleic Acids Res.* **45**, D380–D388 (2017).
2. Vendruscolo, F., Rossi, M.J., Schmidell, W., Ninow, J.L. Determination of oxygen solubility in liquid media. *ISRN Chem. Eng.* **2012**, 601458 (2012).
