# Supplementary material for: Mechanism-based tuning of insect 3,4-dihydroxyphenylacetaldehyde synthase for synthetic bioproduction of benzylisoquinoline alkaloids
Source: Nat Commun. 2019 May 1;10:2015. doi: 10.1038/s41467-019-09610-2 (PMC6494836; doi:10.1038/s41467-019-09610-2)
Supplement: Supplementary file 2 — Peer Review [file 41467_2019_9610_MOESM2_ESM.pdf]

Reviewers' comments:

Reviewer #1 (Remarks to the Author):

Vavricka et. al demonstrates the rational evolution of an insect 3,4-dihydroxyphenylacetaldehyde synthase (DHPAAS) to alter its selectivity, which enables efficient production of THP from L-DOPA. This paper is well organized, and if can push the thebaine production or even downstream opioid production to a new higher level, is a significant finding. However, the author needs to address the following comments to make the story scientifically sound enough:

1) The figures are odd looking and not carefully crafted. For example, in Fig. 2, the structure of reticuline is wrong; and it is not entirely clear what the red color highlighted "NCS->R=H:Norcoclaurine" and ""NMCH* mean, why highlight them, why there's a star; and panel c needs to be better explained in either the main text or figure caption. Overall, all the figures need to be polished and more clearly presented.

2) The major point in engineering the DHPAAS is to more efficient convert L-DOPA to THP is for the synthesis of downstream BIAs, such as thebain as the author demonstrated. However, the author didn't show whether introducing such an enzyme will actually enhance the production of THF-downstream metabolites (considering THF is only the immediate product of the engineered enzyme). It is believed that with the new engineered DHPAAS, the author should be able to achieve a microbial production of thebaine higher than previously reported. Or if not, the author should also discuss other engineering efforts required to further push E. coli thebaine or other downstream BIA production. Also, upon introducing downstream enzymes, will these four mutants exhibit the same trend as in THP production or will change?

3) In Fig. 6 & Fig. 7, please provide error bars on all the data points.

4) It would also be helpful if the author can provide the simulation structure of the mutants, kinetics data on all the four mutants, compared with the wildtype enzyme, to better explain the different in vitro and in vivo performance.

Reviewer #2 (Remarks to the Author):

NCOMMS-18-20028
General comments
The purpose of this work was to construct a synthetic pathway for the production of tetrahydropapaveroline (THP) from L-DOPA by taking advantage of a new recently discovered aromatic aldehyde synthase (AAS) which can on the one hand overcome the metabolic problem of monoamine oxidase and while the natural form generates directly DHPAA, variants obtained by rational engineering could result in a symmetrical formation of dopamine and DHPAA which thereafter gives rise to THP by spontaneous non-enzymatic condensation. The overall work represents a practical demonstration of the D-B-T-L concept in Synthetic Biology. Overall, this work is relevant in the field of Synthetic biology applied to redesigning or constructing new pathways for biochemical manufacture. Although it may deserve interest to the Scientific community, it seems that the paper has been written in a rush (eg many typos remaining! some results are in Discussion section!) and in addition, there are several aspects in this work that need to be modified, corrected or clarified as presented here below.

In Introduction section
The objective of this work does not seem to be clearly justified. The authors claimed that a limitation of the actual pathway is using monoamine oxidase that is highly sensitive to many inhibitors, which would hamper high yield. However, these identified inhibitors (see ref 8) are unlikely to be

produced/present in E coli. On the other hand, this protein, which is likely of mammal origin is an outer membrane –bound mitochondria flavoenzyme. Thus it needs to localize in membrane of E coli that are different from mitochondrial ones, and requires FAD as cofactor. This may pose important problem of expression and localization and thus , of catalytic efficiency. It is therefore advised to engineer a 'soluble 'enzyme

You mentioned the name AAS , but better use DHPAAS instead since AAS is already in use for meaning other enzymes and that can be misleading.
Could you also provide some 'number' in terms of expected titer and yield with these molecules produced by microbes.?

is there also any risk of toxicity by high dosage of these molecule? Is there any data available on that?

In Results section
Design:

• M-Path is of no doubt very useful to mine pathways and enzymes but relies on existing database. While there is no concern about using this tool, emphasizing of its importance to find new enzyme in this particular case sounds odd since the synthesis of DHPAA from L-DOPA by a special AAAD termed DHPAAsynthase was already known and ironically by the first author of this paper (see ref 18 and references within that of 24)

• Caution should be made with this sentence "Although the above mentioned AAS enzymes have been assigned as EC 4.1.1.- by KEGG, many questions still remain about the relatively newly characterized AAS enzymes which are not straightforward to classify due to bifunctional activities. Therefore, AAS enzymes are still under submission to strict databases like BRENDA, and rules for the selection of the best AAS for bioproduction applications should be clearly established. AAS is not yet defined as a new enzyme family and AAS terms is already in use. It may be better to stand with DHPAAS family at this stage.

• The simulation model is very useful. However, the suggested enzyme to catalyze these reactions can be, at variance to MAO, competitively inhibited by the endogenous production of aromatic amino acids tyrosine and phenylalanine that can be substrate of this enzyme. Should you consider this possibility? Do you have any data supporting or contradicting this suggestion? Did you actually check either for feedback inhibition by the end product of enzyme reaction?

• Should you be more explicit with this following sentence: "Later experiments indicated that highly reactive DHPAA is readily depleted in vivo, and after factoring this DHPAA 'drain' into the models, predicted conversions better matched the experimental results"

• I do not see the reason to expand so much on –this following paragraph: "M-path identified 4-HPAAS an as enzyme to produce 4-HPAA, a key intermediate in plant BIA synthesis. Accordingly, we hypothesized that P. somniferum might utilize AAS activity for natural 4-HPAA bioproduction and explored P. somniferum sequences as potential AAS enzymes. While P. somniferum contains multiple sequences annotated as tyrosine decarboxylase (TyDC), most of the TyDC active sites highly resemble that of canonical AAAD. Interestingly, Papaver somniferum TyDC1, which was modeled based on the structure of Sus Scrofa DDC in complex with carbidopa (PDB ID: 1JS3)21, contains a novel isoleucine residue at the position corresponding to AAAD active site His192 (Figure 3, center panel), bringing attention to the this position as an important catalytic residue. Yet, all of the P. somniferum sequences annotated as TyDC contain Tyr350 (P. somniferum TyDC1 numbering, Phe338 in Arabadopsis thaliana

AAS), while a phenylalanine at this position is hypothesized to be essential for plant AAS activity17. In contrast, clear active site differences are seen when comparing putative insect DHPAAS sequences (Figure 3). Therefore, focus was shifted towards insect DHPAAS for the selection of an optimal BIA bioproduction system" since work by Liang et al (ref 24) already emphasized what makes difference between DDC and DPHAAS.

• what is the meaning of this sentence , with respect to the question that follows. "A recent report by Liang et al. analyzed D. melanogaster NP_476592.1, which contains Tyr79-Tyr80 where Tyr79 is conserved in DDC24. While D. melanogaster also contains a distinct DHPAAS sequence NP_724162 containing both DHPAAS specific residues Phe79 and Tyr80, characterization of NP_724162 has not yet been reported"

Build

• Was the synthetic genes of B.mori codon optimized for E coli?

• There is no data that shows that the Asn192His and Phe79Tyr-Ty80Phe variants have a a mixture of DHPAAS and DDC activities. Should it be those presented in Table of Fig.8?

• as you expressed the protein in vitro, it would be interesting to have some kinetic data such Km / kcat of each these variants to L-DOPA and potential feedback effect by THP or end product, which would help in refining the model you have presented above.

Test

• the X-scale for H202 production does not match that for DOPA, THP etc production. Why

• In the figure showing DA production, why there is DA in sample at time 0 for WT ; Should L-DOPA contaminated with DA?

• Please rewrite this :" As predicted, overall DHPAA production was highest using the wild-type enzyme, second highest with the Tyr79Phe-Phe80Tyr variant, third highest with the Asn192His variant, and lowest when using the Tyr79Phe-Phe80Tyr-Asn192His variant"

• sentence: "In the presence of ascorbate, DHPAA was sometimes measured to exceed initial substrate concentration, likely due to the lower stability of DHPAA in standard curve solutions of methanol with no ascorbate; This could be eventually quoted in M&LM but as you are aware ofthis, why did you not make the correction?

• This sentence : Fortunately, after changing to M9 minimal medium, THP production increased significantly (Figure 7)… Changing to what.?

Discussion

• Should you consider that the difference between in vitro and in vivo THP production by asn192his DHPAAS variant due to high production of H202, since in vitro THP title is high with this variant only in the presence of ascorbate!

• H202 is likely a real problem in this production. You should consider overexpressing katE encoding the catalase II that mainly takes over excess of H202

• data on Figure 8 (and corresponding description) should be transferred into Results

• How the calculation was made in table of Fig 8 ! I would expect a correspondence between H202 activity and DHPAA contribution since DDC does not generate H202

• last part of Discussion sounds to be 'out ' of this work
M& M

• In vivo production: should it be so precise with the time, (1 h13 post induction, 12 h52 after addition..)

• induction with 0,97 mM IPTG? Did you check the optimal concentration for induction? Often to high IPTG is not good

Reviewer #3 (Remarks to the Author):

I take the liberty to reply to te editor only (pls. see below).

[Editor: In the Remarks to Editor section this reviewer states that citation of ref 19 is not an obvious choice. Ref 19 proposes a multicriteria analysis which combines green chemistry principles, techno-economic analysis and some elements of environmental life-cycle assessment (LCA). But these elements didn't apply to this study. The only links are the application of Monte-Carlo simulation (which is broadly applied for probabilistic analyses including uncertainty analysis) and the application of the method to bio-based processes. Please check the references for accuracy and remove ref 19 if it's not the appropriate citation.]

Reviewer #4 (Remarks to the Author):

"Mechanism Based Tuning of Insect 3,4-Dihydroxyphenylacetaldehyde Synthase for Synthetic Bioproduction of Tetrahydropapaveroline", Vavricka et. al

The manuscript by Vavricka and colleagues describes the engineering of a novel metabolic pathway for the production tetrahydropapaveroline, an opioid analgesic precursor.

Given the emphasis placed on the Design-Build-Test-Learn approach taken, I think it should be made clearer in the Results section that you are using dynamical modelling to select the pathway topology. Perhaps separate out the M-path results from the dynamical modelling to make this clearer to the reader.

Page 5 and Figure 1 caption: "Later experiments indicated that highly reactive DHPAA is readily depleted in vivo, and after factoring this DHPAA 'drain' into the models, predicted conversions better matched the experimental results." I could not find evidence of this in either the main text or the supplementary methods. In fact, it seems that nowhere are the models compared to data. How much better was the agreement? Was a statistical test performed, such as a likelihood ratio test, to demonstrate a better fit despite the additional parameter?

There are a number of arbitrary choices made regarding parameter values in table S1. While this is unavoidable given current knowledge, some study of these assumptions on the conclusions made in figure S3 should be performed. How robust is this result?

Another issue is how parameter space is sampled. Often for these types of systems, because we really

don’t know what order of magnitude the parameters are, a uniform on the log scale is preferable. Did the authors look into this? Would this affect the choice of pathway topology?

The code used to generate the results should be made available either as a supplementary folder or (ideally) in a repository such as GitHub. For reproducibility purpose this should be set up so that someone can easily run the code and reproduce the results in Figure S3.

MODELLER tool needs a reference and perhaps a short explanation on page 5.

**Response to Reviewer Comments**

**Reviewer #1 (Remarks to the Author):**

Vavricka et. al demonstrates the rational evolution of an insect 3,4-dihydroxyphenylacetaldehyde synthase (DHPAAS) to alter its selectivity, which enables efficient production of THP from L-DOPA. This paper is well organized, and if can push the thebaine production or even downstream opioid production to a new higher level, is a significant finding. However, the author needs to address the following comments to make the story scientifically sound enough:
***Response:*** *The positive remarks by the Reviewer are greatly appreciated. To satisfy the recommendation of the Reviewer, we have worked hard very hard to expand downstream BIA production of reticuline from THP as explained in more detail below.*

1) The figures are odd looking and not carefully crafted. For example, in Fig. 2, the structure of reticuline is wrong; and it is not entirely clear what the red color highlighted "NCS->R=H:Norcoclaurine" and ""NMCH* mean, why highlight them, why there's a star; and panel c needs to be better explained in either the main text or figure caption. Overall, all the figures need to be polished and more clearly presented.
***Response:*** *Thank you for carefully checking the figures and manuscript. According to the Reviewer's advice, all of the Figures have been carefully revised. To improve the clarity of Fig. 2, a separate illustration of the norcoclaurine pathway is included, the Fig. 2 legend is improved, and the structure of reticuline has been corrected. Figure 2C has been completely revised as a separate figure to address the comments of Reviewer #4.*

2) The major point in engineering the DHPAAS is to more efficient convert L-DOPA to THP is for the synthesis of downstream BIAs, such as thebain as the author demonstrated. However, the author didn't show whether introducing such an enzyme will actually enhance the production of THF-downstream metabolites (considering THF is only the immediate product of the engineered enzyme). It is believed that with the new engineered DHPAAS, the author should be able to achieve a microbial production of thebaine higher than previously reported. Or if not, the author should also discuss other engineering efforts required to further push E. coli thebaine or other downstream BIA production. Also, upon introducing downstream enzymes, will these four mutants exhibit the same trend as in THP production or will change?
***Response:*** *These important recommendations are very helpful for improving the manuscript. The option of "other downstream BIA production" is appreciated. Although our laboratory currently has no permission to produce highly controlled compounds like thebaine or active pain medicines, we were able to satisfy the request for other downstream BIA production within a reasonable time period for revision.*

*Following this recommendation, the downstream BIA product reticuline was successfully produced in E. coli using our DHPAAS production system. After the*

*addition of 6-OMT, CNMT and 4-OMT (now explained in the revised Figure 2 legend), reticuline was produced via all four of our DHPAAS variants. Consistent with our in vivo THP production results, Phe79Tyr-Tyr80Phe DHPAAS was also the most efficient reticuline producer. These results are now presented with error bars as Figure 11.*

3) In Fig. 6 & Fig. 7, please provide error bars on all the data points.
***Response:*** *To address this important comment, the previous in vitro and in vivo bioproduction experiments have been carefully re-performed in triplicate. Fortunately, just enough frozen DHPPAS protein stocks were available to finish in vitro production replicates and additionally requested enzyme kinetic experiments. DHPAAS kinetic experiments were performed via measurement of $H_2O_2$ product and these results now appear as revised Figure 7. The new kinetic analysis of $H_2O_2$ production is consistent with the $H_2O_2$ production results shown in the previous version of Figure 6, and therefore the relevant panel has been removed. Revised in vivo bioproduction results are now presented as Figure 10.*

4) It would also be helpful if the author can provide the simulation structure of the mutants, kinetics data on all the four mutants, compared with the wildtype enzyme, to better explain the different in vitro and in vivo performance.
***Response:*** *As requested, structural analysis of all variants is now included to provide more insight into the mechanism of DHPAAS mechanism switching. All structures are shown in the revised Figure 4. In addition, kinetic analysis of all DHPAAS variants in comparison to wild-type DHPAAS is included. All of this new kinetic data is consistent with our model of DHPAAS engineering.*

**Reviewer #2 (Remarks to the Author):**

**NCOMMS-18-20028**
**General comments**
The purpose of this work was to construct a synthetic pathway for the production of tetrahydropapaveroline (THP) from L-DOPA by taking advantage of a new recently discovered aromatic aldehyde synthase (AAS) which can on the one hand overcome the metabolic problem of monoamine oxidase and while the natural form generates directly DHPAA, variants obtained by rational engineering could result in a symmetrical formation of dopamine and DHPAA which thereafter gives rise to THP by spontaneous non-enzymatic condensation. The overall work represents a practical demonstration of the D-B-T-L concept in Synthetic Biology. Overall, this work is relevant in the field of Synthetic biology applied to redesigning or constructing new pathways for biochemical manufacture.
Although it may deserve interest to the Scientific community, it seems that the paper has been written in a rush (eg many typos remaining! some results are in

Discussion section!) and in addition, there are several aspects in this work that need to be modified, corrected or clarified as presented here below.
***Response:*** *Many thanks to the Reviewer for believing that the manuscript successfully applies the D-B-T-L concept towards the construction of bioproduction pathways. We apologize for the many required revisions due to pressure to quickly submit this competitive manuscript. However, extensive major revisions have been carefully carried out to ensure that the manuscript can now be acceptable for publication.*

In Introduction section
The objective of this work does not seem to be clearly justified. The authors claimed that a limitation of the actual pathway is using monoamine oxidase that is highly sensitive to many inhibitors, which would hamper high yield. However, these identified inhibitors (see ref 8) are unlikely to be produced/present in E coli. On the other hand, this protein, which is likely of mammal origin is an outer membrane –bound mitochondria flavoenzyme. Thus it needs to localize in membrane of E coli that are different from mitochondrial ones, and requires FAD as cofactor. This may pose important problem of expression and localization and thus, of catalytic efficiency. It is therefore advised to engineer a 'soluble 'enzyme
***Response:*** *We apologize for not sufficiently justifying the work. The description of MAO inhibition has been removed from the second paragraph of the introduction, and has been replaced with the Reviewer's improved description of MAO as a membrane bound flavoenzyme.*

You mentioned the name AAS, but better use DHPAAS instead since AAS is already in use for meaning other enzymes and that can be misleading. Could you also provide some 'number' in terms of expected titer and yield with these molecules produced by microbes?
***Response:*** *DHPAAS is now used in reference to the insect protein throughout the entire manuscript. The computational models predicted that DHPAAS mediated pathways have potential to surpass MAO mediated pathways. The MAO mediated pathway has resulted in titers of 1 mM THP (reference 7). Therefore, we expect that after optimization, the DHPAAS mediated pathway has potential to produce THP at a titer of above 1 mM. This is now stated in the description of pathway prediction results.*

is there also any risk of toxicity by high dosage of these molecule? Is there any data available on that?
***Response:*** *This is a very important point. The toxicity of DHPAA and THP are described in references 19 and 6, respectively. Therefore, flux from DHPAA to THP, and from THP to less-toxic retculine, should be optimized. Furthermore, methods for reduction of $H_2O_2$ should be present in an optimal bioproduction system. These points are now included in the discussion section.*

In Results section
Design:

• M-Path is of no doubt very useful to mine pathways and enzymes but relies on existing database. While there is no concern about using this tool, emphasizing of its importance to find new enzyme in this particular case sounds odd since the synthesis of DHPAA from L-DOPA by a special AAAD termed DHPAA synthase was already known and ironically by the first author of this paper (see ref 18 and references within that of 24)

***Response:*** *The Reviewer's point is very important. In order to carefully address this issue, a statement in the first paragraph of the results has been revised as follows:*

*"Although authors were aware of DHPAAS, this example of enzyme selection illustrates the importance of updating enzyme databases to predict recently characterized enzymes, as many new functions are continuously being discovered from nature and also created artificially through enzyme engineering[16]."*

• Caution should be made with this sentence "Although the above mentioned AAS enzymes have been assigned as EC 4.1.1.- by KEGG, many questions still remain about the relatively newly characterized AAS enzymes which are not straightforward to classify due to bifunctional activities. Therefore, AAS enzymes are still under submission to strict databases like BRENDA, and rules for the selection of the best AAS for bioproduction applications should be clearly established. AAS is not yet defined as a new enzyme family and AAS terms is already in use. It may be better to stand with DHPAAS family at this stage.

***Response:*** *To carefully address this important comment, the entire paragraph has been rewritten. Throughout the entire revised manuscript, DHPAAS is no longer explicitly defined as a member of the AAS group.*

• The simulation model is very useful. However, the suggested enzyme to catalyze these reactions can be, at variance to MAO, competitively inhibited by the endogenous production of aromatic amino acids tyrosine and phenylalanine that can be substrate of this enzyme. Should you consider this possibility? Do you have any data supporting or contradicting this suggestion? Did you actually check either for feedback inhibition by the end product of enzyme reaction?

***Response:*** *Thank you for the stimulating suggestions. Fortunately, no activity was observed toward tyrosine or phenylalanine by the Phe79Tyr-Tyr80Phe-Asn192His variant.*

*The Asn192His and Phe79Tyr-Tyr80Phe variants could not be additionally tested at this time as all of their enzyme stocks were completely consumed to finish the requested replicate and kinetic experiments. The remaining wild-type DHPAAS stock was used to do a preliminary test of inhibition by dopamine and DHPAA. Mild inhibition of the wild-type enzyme and Phe79Tyr-Tyr80Phe-Asn192His variant was observed when using dopamine and DHPAA in the 100 µM range. THP did not appear to inhibit the Phe79Tyr-Tyr80Phe-Asn192His variant. However, high concentration*

*product standards are dissolved in MeOH or MeCN, which complicates the inhibition analysis. Due to these issues, it was not possible to complete a comprehensive analysis of DHPAA inhibitors in such a short time.*

*Based on our results that address this important comment, the following paragraph has been added to the discussion:*

*"As no DHPAAS activity was observed towards tyrosine or phenylalanine by the Phe79Tyr-Tyr80Phe-Asn192His variant, competitive inhibition by aromatic amino acids may not be a major consideration for DHPAAS mediated biosynthesis. However, a preliminary analysis indicates mild feedback inhibition of DHPAAS by dopamine and DHPAA, and possible substrate inhibition at high concentrations of L-DOPA. These factors, together with the low measured Km of B. mori DHPAAS, may help to explain why high concentration L-DOPA was not completely consumed during in vivo bioproduction experiments. It is also possible that DHPAAS is inhibited by unknown growth medium components. Therefore, substrate specificity and inhibition should be comprehensively characterized as learning data for enzyme improvement."*

• Should you be more explicit with this following sentence: "Later experiments indicated that highly reactive DHPAA is readily depleted in vivo, and after factoring this DHPAA 'drain' into the models, predicted conversions better matched the experimental results"
***Response:*** *To address this important comment and also a related comment of Reviewer #4 the relevant text has been revised to the following:*

*"Later in vitro and in vivo tests suggested that highly reactive DHPAA may be depleted by reaction with competing nucleophiles present within cells or in the growth medium. Including this DHPAA 'drain' in dynamic models resulted in slightly lower THP yields (Figure 3), indicating a better match to experiential yields. However, many diverse variables, including buffer composition of growth medium, pH, temperature, potential inhibitors and metabolic flux, should also be considered as learning data for the improvement of THP yields."*

• I do not see the reason to expand so much on –this following paragraph: "M-path identified 4-HPAAS an as enzyme to produce 4-HPAA, a key intermediate in plant BIA synthesis. Accordingly, we hypothesized that P. somniferum might utilize AAS activity for natural 4-HPAA bioproduction and explored P. somniferum sequences as potential AAS enzymes. While P. somniferum contains multiple sequences annotated as tyrosine decarboxylase (TyDC), most of the TyDC active sites highly resemble that of canonical AAAD. Interestingly, Papaver somniferum TyDC1, which was modeled based on the structure of Sus Scrofa DDC in complex with carbidopa (PDB ID: 1JS3)21, contains a novel isoleucine residue at the position corresponding to AAAD active site His192 (Figure 3, center panel), bringing attention to the this position as an important catalytic residue. Yet, all of the P. somniferum sequences annotated as TyDC contain Tyr350 (P. somniferum TyDC1 numbering, Phe338 in

Arabadopsis thaliana AAS), while a phenylalanine at this position is hypothesized to be essential for plant AAS activity17. In contrast, clear active site differences are seen when comparing putative insect DHPAAS sequences (Figure 3). Therefore, focus was shifted towards insect DHPAAS for the selection of an optimal BIA bioproduction system" since work by Liang et al (ref 24) already emphasized what makes difference between DDC and DPHAAS.
***Response:*** *We agree with the Reviewer that the manuscript is much better after extensively shortening this section.*

• what is the meaning of this sentence , with respect to the question that follows. "A recent report by Liang et al. analyzed D. melanogaster NP_476592.1, which contains Tyr79-Tyr80 where Tyr79 is conserved in DDC24. While D. melanogaster also contains a distinct DHPAAS sequence NP_724162 containing both DHPAAS specific residues Phe79 and Tyr80, characterization of NP_724162 has not yet been reported"
Build
***Response:*** *We thank the reviewer for bringing attention this section. To shift more attention to the current study, mention of the work by Liang et al. has been removed from this paragraph.*

• Was the synthetic genes of B. mori codon optimized for E coli?
***Response:*** *The native B. mori DHPAAS sequence was used in all constructs for this study. This is now clarified in the methods section.*

• There is no data that shows that the Asn192His and Phe79Tyr-Ty80Phe variants have a a mixture of DHPAAS and DDC activities. Should it be those presented in Table of Fig.8?
***Response:*** *Production of both DHPAA and dopamine by the Asn192His and Phe79Tyr-Tyr80Phe variants is shown in the in vivo and in vitro production experiments. However, this was not made explicit in the previous version and we apologize for that. To clarify this point, Figure 8 has been improved as revised Figure 9, which now lists measured DHPAAS and DDC activity rates. This is further explained in the related two comments below.*

• as you expressed the protein in vitro, it would be interesting to have some kinetic data such Km / kcat of each these variants to L-DOPA and potential feedback effect by THP or end product, which would help in refining the model you have presented above.
Test
***Response:*** *Km and kcat values for all DHPAAS variants are now presented in the revised Figure 7. As mentioned above, some dopamine and DHPAAS inhibition was observed in preliminary experiments, and this is now addressed in the discussion section.*

• the X-scale for H202 production does not match that for DOPA, THP etc production. Why

***Response:*** *The $H_2O_2$ production experiment was performed as a fluorescent assay, separate from the mass spectrometric detection of aromatic products. This experiment has been replaced by the requested DHPAAS kinetic analysis with the X-axis representing [L-DOPA].*

• In the figure showing DA production, why there is DA in sample at time 0 for WT; Should L-DOPA contaminated with DA?
***Response:*** *Although it is difficult to see, the first measurement of in vitro production was taken 9 min. after starting the reaction. To clarify this problem, the measurement times are now listed in the Figure legend.*

• Please rewrite this :" As predicted, overall DHPAA production was highest using the wild-type enzyme, second highest with the Tyr79Phe-Phe80Tyr variant, third highest with the Asn192His variant, and lowest when using the Tyr79Phe-Phe80Tyr-Asn192His variant"
***Response:*** *The relevant text has been rewritten as requested.*

• sentence: "In the presence of ascorbate, DHPAA was sometimes measured to exceed initial substrate concentration, likely due to the lower stability of DHPAA in standard curve solutions of methanol with no ascorbate; This could be eventually quoted in M&LM but as you are aware of this, why did you not make the correction?
***Response:*** *Fortunately, this issue has been greatly improved in the revised triplicate in vitro data the relevant description is no longer necessary.*

• This sentence : Fortunately, after changing to M9 minimal medium, THP production increased significantly (Figure 7)… Changing to what.?
***Response:*** *We apologize for the confusing statement. The relevant section has been revised as follows:*

*"Fortunately, after changing the growth medium from LB to M9 minimal medium, THP production increased significantly (Figure 10)."*

Discussion
• Should you consider that the difference between in vitro and in vivo THP production by asn192his DHPAAS variant due to high production of H202, since in vitro THP title is high with this variant only in the presence of ascorbate!
***Response:*** *To address this very important comment the following sentence has been added to the in vitro results section:*

"*In vitro* THP yields improved significantly after ascorbate supplementation to overcome degradation of products by $H_2O_2$ mediated oxidation."

• H202 is likely a real problem in this production. You should consider overexpressing katE encoding the catalase II that mainly takes over excess of H202

***Response:*** *Thank you for the great suggestion, which has been added to the discussion.*

• data on Figure 8 (and corresponding description) should be transferred into Results

***Response:*** *DHPAAS and DDC activities are now calculated based on $H_2O_2$ production rates and in vitro dopamine production rates, respectively. Accordingly, Figure 8 has been moved to the in vitro results section as Figure 9, directly following the presentation of enzyme kinetics and in vitro bioproduction. This arrangement, suggested by the Reviewer, is more logical.*

• How the calculation was made in table of Fig 8 ! I would expect a correspondence between H202 activity and DHPAA contribution since DDC does not generate H202

***Response:*** *The previous estimates of DHPAAS to DDC activity ratios were based on the first in vivo data measurements. As the in vivo conditions have more variables, we now follow the Reviewer's suggestion to use $H_2O_2$ production as a measure of DHPAAS activity. Moreover, the revised $H_2O_2$ assay was more carefully controlled with measurements of the initial reaction rate taken every 20 seconds. For the DDC activity estimate, we now use the measurement of the stable intermediate dopamine at the first time point of the in vitro assay (9 minutes). The revised data is now presented as Figure 9.*

• last part of Discussion sounds to be 'out ' of this work

M& M

***Response:*** *The relevant content of the last paragraph is about expanding DBTL cycles to new related cycles, but it has been extensively shortened to two sentences.*

*The entire discussion has now been reorganized to focus on two major types of learning for improving THP and reticuline yields: learning for enzyme improvement, and learning for metabolic flux improvement.*

• In vivo production: should it be so precise with the time, (1 h13 post induction, 12 h52 after addition..)

***Response:*** *In vivo production times are now more clearly specified in relation to substrate addition.*

• induction with 0.97 mM IPTG? Did you check the optimal concentration for induction? Often to high IPTG is not good

***Response:*** *This helpful suggestion is also appreciated. After reading the comments, we tested reticuline production with 0.1, 0.5 and 1 mM IPTG, but the THP and reticuline titers were similar in all conditions.*

**Reviewer #3 (Remarks to the Author):**

I take the liberty to reply to te editor only (pls. see below).

[Editor: In the Remarks to Editor section this reviewer states that citation of ref 19 is not an obvious choice. Ref 19 proposes a multicriteria analysis which combines green chemistry principles, techno-economic analysis and some elements of environmental life-cycle assessment (LCA). But these elements didn't apply to this study. The only links are the application of Monte-Carlo simulation (which is broadly applied for probabilistic analyses including uncertainty analysis) and the application of the method to bio-based processes. Please check the references for accuracy and remove ref 19 if it's not the appropriate citation.]
***Response:*** *We apologize for the poor citation and thank the reviewer for pointing this out. To ensure that no inappropriate references are cited, the Supplementary Information is now only cited here. Furthermore, the Supplementary Information has been updated to include more details about the Monte Carlo method, and the source code will be uploaded to Github upon acceptance of the manuscript.*

**Reviewer #4 (Remarks to the Author):**

"Mechanism Based Tuning of Insect 3,4-Dihydroxyphenylacetaldehyde Synthase for Synthetic Bioproduction of Tetrahydropapaveroline", Vavricka et. Al

The manuscript by Vavricka and colleagues describes the engineering of a novel metabolic pathway for the production tetrahydropapaveroline, an opioid analgesic precursor.

Given the emphasis placed on the Design-Build-Test-Learn approach taken, I think it should be made clearer in the Results section that you are using dynamical modelling to select the pathway topology. Perhaps separate out the M-path results from the dynamical modelling to make this clearer to the reader.
***Response:*** *To clarify this important point, the M-path and dynamic modeling results are now presented as two separate figures in the revised manuscript. Furthermore, the dynamic modeling results are now presented more clearly as box plots in the main text.*

Page 5 and Figure 1 caption: "Later experiments indicated that highly reactive DHPAA is readily depleted in vivo, and after factoring this DHPAA 'drain' into the models, predicted conversions better matched the experimental results." I could not find evidence of this in either the main text or the supplementary methods. In fact, it seems that nowhere are the models compared to data. How much better was the

agreement? Was a statistical test performed, such as a likelihood ratio test, to demonstrate a better fit despite the additional parameter?

***Response:*** *Apologies for not explaining this more clearly and over-emphasizing DHPAA 'drain'. As shown below, after adding this 'drain' into the calculations, predicted yields decreased slightly which may help to explain the lower experimental yields: in vitro yields were around 20% and in vivo yields were even lower.*

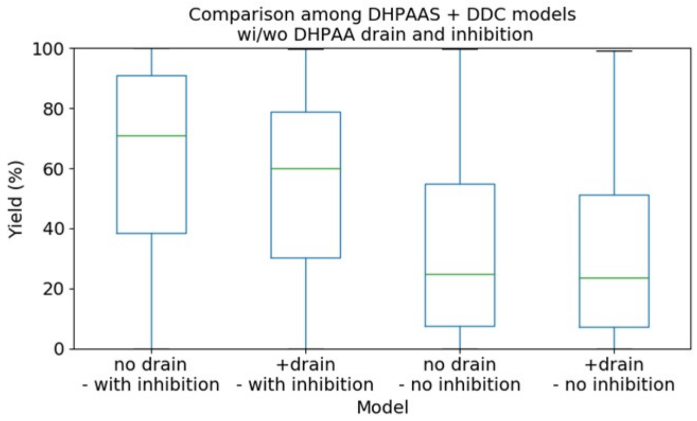


*In order to avoid any potentially misleading statements, DHPAAS 'drain' has been emphasized less in the following revision to the respective text:*

*"Later in vitro and in vivo tests suggested that highly reactive DHPAA may be depleted by reaction with competing nucleophiles present within cells or in the growth medium. Including this DHPAA 'drain' in dynamic models resulted in slightly lower THP yields (Figure 3), indicating a better match to experiential yields. However, many diverse variables, including buffer composition of growth medium, pH, temperature, potential inhibitors and metabolic flux, should also be considered as learning data for the improvement of THP yields"*

There are a number of arbitrary choices made regarding parameter values in table S1. While this is unavoidable given current knowledge, some study of these assumptions on the conclusions made in figure S3 should be performed. How robust is this result?

***Response:*** *As the reviewer pointed out, data for required parameters is indeed insufficient, and as a result, some parameters are set within arbitrary ranges. MAO and DHPAAS models including feedback inhibition and DHPAA 'drain' were also tested with arbitrary parameters set in the log scale. As described below, this updated analysis still resulted in higher median THP yield predicted for the DHPAAS mediated pathway. In this regard the overall results are robust.*

Another issue is how parameter space is sampled. Often for these types of systems, because we really don't know what order of magnitude the parameters are, a uniform on the log scale is preferable. Did the authors look into this? Would this affect the choice of pathway topology?

***Response:*** *To address the important point of the Reviewer, the DHPAAS-DDC and MAO simulations with feedback inhibition and DHPAA drain were re-performed with parameters in log scales. The range of the log scale is much larger and includes more suboptimal parameters; therefore the resulting THP yields were much lower. Despite this, the median THP production still remained higher for the DHPAAS-DDC pathway (0.00375%) relative to that of the MAO pathway (0.000076%). As the THP yield distributions are not normal, we take the median score as the most important benchmark.*

The code used to generate the results should be made available either as a supplementary folder or (ideally) in a repository such as GitHub. For reproducibility purpose this should be set up so that someone can easily run the code and reproduce the results in Figure S3.
***Response:*** *As requested by the referee, we agree to upload the python source code to GitHub (https://www.github.com/yukuriya3/For_THP_pro_path_select) upon acceptance of the manuscript.*

MODELLER tool needs a reference and perhaps a short explanation on page 5.
***Response:*** *A brief description of MODELLER and Chimera use and the relevant citations are now included on page 5 and also in the methods.*

Reviewers' comments:

Reviewer #1 (Remarks to the Author):

This revised manuscript has been quite improved, and has included a lot more data. However, I still feel this paper is not well presented, and I am also not sure if this story is novel enough for this journal.

First, figure 1, it is still very difficult to understand the purpose of this figure. If the author wants to present this D-B-T-L as a new methodology, I won't really agree with that. Many previous stories have well demonstrated such workflow, for example, the ref 27. I don't think this is the major selling point of this story. Rather, the engineered bifunctional DHPAAS and a potential high producer of reticuline will be more appealing.

However, the reticuline production is ~0.2uM, which is ~ 66mg/L. The author demonstrates that this is up to date the highest, which is not true. The recent published paper on BIA analogs bi-production in E. coli (https://doi.org/10.1038/s41598-018-26306-7) has presented that production of (S)-reticuline is up to 150mg/L in the engineered E. coli strain, which should be a better comparison to this paper rather than ref 27 using yeast as the producer. 66mg/L is thus not an appealing yield to be achieved from E. coli.

In addition, it seems an easy experiment for the author to coexpress the Phe79Tyr-Tyr80Phe-Asn192His variant and wildtype DHPAAS, the most efficient DHPAA and DA producing enzyme, respectively.

Reviewer #2 (Remarks to the Author):

The revised ms have been greatly improved and it is in a better shape for publication in Nat Comm Yet, there is still some substantial inconsistencies or mistakes that shall be corrected before its acceptance
page 1, line 46: I would remove this term 'Fortunately'!
page 2, line 59 and 63: the term 'yield ' is not used appropriately. It is rather here 'titer' or production. In addition, this titer is linked to the concentration of DOPA that is provided to the cells! Therefore, I wonder if these terms are useful!
page 3, line 106: 'of' is missing at the end of the sentence
page 8, line 269: 'make' is missing in this sentence
page 11, figure 8: You should precise in the figure how much DOPA was added in the medium
page 12, The table must be numbered
page 13, figure 10: You should indicate how much DOPA was added in the culture
Page 13, line 435 and after: an indication about the expected yield and the actual yield of THF from DOPA would be relevant, in order to better appreciate the efficiency of the synthetic pathway.
Page 14, line 478-479: ‵high concentration ‵.. of DOPA not completely consumed? Do you mean that if DOPA is very low, the consumption would be complete?
Page 18; line 642: Why do you add PLP and not simply pyridoxine to the culture? Is this addition of PLP necessary?

Reviewer #4 (Remarks to the Author):

My specific comments have now been addressed. The dynamical modelling has been given more emphasis and the robustness has been explored and verified.

**Response to Reviewer Comments on:**
**Mechanism Based Tuning of Insect 3,4-Dihydroxyphenylacetaldehyde Synthase for Synthetic Bioproduction of Benzylisoquinoline Alkaloids**

## Reviewer #1 (Remarks to the Author):

This revised manuscript has been quite improved, and has included a lot more data. However, I still feel this paper is not well presented, and I am also not sure if this story is novel enough for this journal.

***Response:*** *We thank the reviewer for believing that the manuscript is improved. Great efforts have been taken to improve the presentation and story even further based on the Reviewer's new requests.*

First, figure 1, it is still very difficult to understand the purpose of this figure. If the author wants to present this D-B-T-L as a new methodology, I won't really agree with that. Many previous stories have well demonstrated such workflow, for example, the ref 27. I don't think this is the major selling point of this story. Rather, the engineered bifunctional DHPAAS and a potential high producer of reticuline will be more appealing.

***Response:*** *To directly address this important comment, the 'DBTL' description has been deleted from the abstract, mention of 'DBTL' has been shortened and less emphasized in the introduction section, and Figure 1 has been totally removed. In combination with the relevant comments on reticuline improvement, a simplified version of the 'DBTL' cycle is included in the final figure. 'DBTL' is only mentioned once in the discussion text to make sure it is not overemphasized.*

However, the reticuline production is ~0.2uM, which is ~ 66mg/L. The author demonstrates that this is up to date the highest, which is not true. The recent published paper on BIA analogs bi-production in E. coli (https://doi.org/10.1038/s41598-018-26306-7) has presented that production of (S)-reticuline is up to 150mg/L in the engineered E. coli strain, which should be a better comparison to this paper rather than ref 27 using yeast as the producer. 66mg/L is thus not an appealing yield to be achieved from E. coli.

***Response:*** *We are happy that the reviewer has pointed out the success of our co-author Dr. Hiromichi Minami's study, which we cite in the revision. This project actually began in collaboration with Dr. Minami when MAO was identified as a bottleneck in his THP production system, and this initiated our search for alternatives enzymes to MAO, leading to our engineering of DHPAAS.*

*The cited works by our co-author Dr. Minami use a complete pathway from glucose to produce (S)-reticuline. This MAO dependent system includes norcoclaurine synthase*

*(NCS) to selectively produce (S)-THP leading to only (S)-reticuline in E. coli grown on rich TB medium. Our system is quite unique in that it focuses on the engineering of a single enzyme, DHPAAS, for more direct production of (R-S)-THP from L-DOPA in M9 minimal medium. Our system is currently inhibited by rich medium and results in ~20 fold lower cell densities, which are challenges for reticuline titers. The previous version of Dr. Minami's system actually separated THP production and reticuline production in two separate cell cultures, highlighting the challenge of producing both THP and reticuline within a single cell (see References 6 and 7).*

*It is somewhat difficult to directly compare Dr. Minami's completed system with our newly developed DHPAAS system at this stage. When using our Asn192His and Phe79Tyr-Tyr80Phe-Asn192His DHPAAS variants, reticuline titers surpassed THP titers suggesting higher conversion of THP to reticuline; this point has been added to the reticuline results. However, we have still made many attempts to improve the current study while using the previous system of Dr. Minami as a benchmark.*

*Immediately after receiving the Reviewer's comments, we spent 4 weeks continuously constructing and testing new reticuline producers with various gene combinations and varying amounts and timing of IPTG, growth medium, $O_2$, glucose, SAM, PLP, and ascorbate. The request to construct and test strains with an additional DHPAAS-Phe79Tyr-Tyr80Phe-Asn192His gene, as well as strains containing an additional DDC gene are detailed below.*

In addition, it seems an easy experiment for the author to coexpress the Phe79Tyr-Tyr80Phe-Asn192His variant and wildtype DHPAAS, the most efficient DHPAA and DA producing enzyme, respectively.

***Response:*** *The reviewer's recommendations are appreciated; accordingly the following BL21(DE3) strains were rapidly constructed and analyzed continuously over the past 4 weeks:*

***1)*** *3 plasmid system with wild-type DHPAAS in pTrcHis2B, DHPAAS-Phe79Tyr-Tyr80Phe-Asn192His in pE-SUMO, and 3 methyltransferases in pACYC184.*

***2)*** *Wild-type DHPAAS in pTrcHis2B, and P. putida DDC together with 3 methyltransferases in pACYC184.*

***3)*** *DHPAAS-Phe79Tyr-Tyr80Phe in pTrcHis2B, and P. putida DDC together with 3 methyltransferases in pACYC184.*

*In the new experiments, the addition of DHPAAS-Phe79Tyr-Tyr80Phe-Asn192His together with wild-type DHPAAS (**1**) resulted in better reticuline titers relative to that of the previous best DHPAAS-Phe79Tyr-Tyr80Phe only system. However, the addition*

*of wild-type P. putida DDC resulted in increased dopamine and THP production together with lower reticuline titers. This further indicates that a bottleneck from THP to reticuline is holding back the titers.*

*Further attempts to follow the conditions of our co-author Hiromichi Minami, by lowering IPTG and attempting higher cell densities, did not increase reticuline titers of our bioproducers. Use of minimal medium with DHPAAS was critical as TB and LB repeatedly inhibited THP and reticuline production. Reticuline titers were limited by low cell densities in minimal medium and apparent conversion/degradation of reticuline, which is still being investigated. The below LC-MS results show decreasing reticuline over time in a sample that was prepared by filtration of culture medium and dilution into 50% MeCN supplemented with 100 μM ascorbate.*

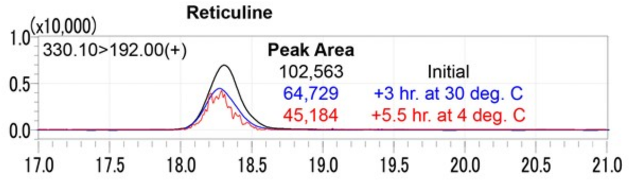


*In order to provide more insight into the bottleneck and to demonstrate a thorough response the Reviewer's important comment, an expanded metabolomics analysis including 115 central metabolites and reticuline intermediates was carried out. Key metabolites involved in the reticuline bottleneck are described in the revision and presented as a new final figure as shown below.*

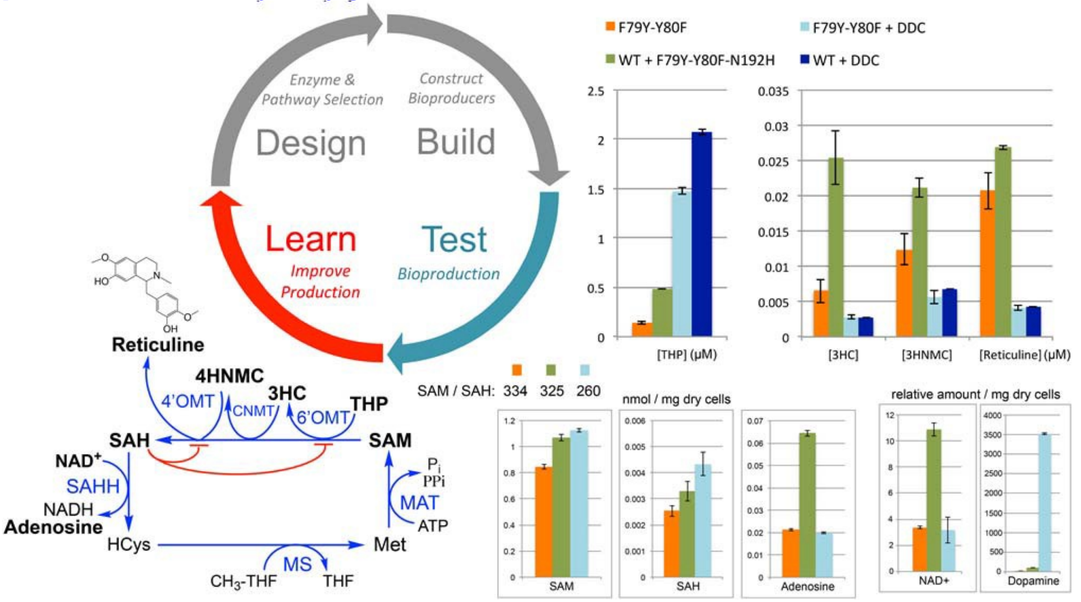


*The expanded metabolomics results indicate that the THP to reticuline bottleneck is pronounced at the 6'OMT step, and that this bottleneck is the major barrier of reticuline production in our system.*

*In summary, we were able to achieve increased THP titers, some relief of the*

*bottleneck from THP to reticuline, and new insights into the reticuline bottleneck. We appreciate the reviewer's suggestions, which have helped to improve this study, and we hope the reviewer can also respect our hard work to follow their increasing instructions within such a limited time frame of a second round revision.*

Reviewer #2 (Remarks to the Author):

The revised ms have been greatly improved and it is in a better shape for publication in Nat Comm

Yet, there is still some substantial inconsistencies or mistakes that shall be corrected before its acceptance

***Response:*** *The constructive comments are greatly appreciated, and we apologize for the inconsistencies and mistakes, which have been carefully corrected.*

page 1, line 46: I would remove this term 'Fortunately'!

***Response:*** *'Fortunately' has been removed here.*

page 2, line 59 and 63: the term 'yield ' is not used appropriately. It is rather here 'titer' or production. In addition, this titer is linked to the concentration of DOPA that is provided to the cells! Therefore, I wonder if these terms are useful!

***Response:*** *Thank you for the important comment. Improper use of 'yield' has been replaced with 'titer', 'production' and 'level' as appropriate throughout the manuscript.*

page 3, line 106: 'of' is missing at the end of the sentence

***Response:*** *We apologize for the mistake and have added the necessary 'of'.*

page 8, line 269: 'make' is missing in this sentence

***Response:*** *The reviewer must be talking in reference to the hydrogen bond. We have clarified this part by revising as: '192 residue can* ***form*** *a hydrogen bond'.*

page 11, figure 8: You should precise in the figure how much DOPA was added in the medium

***Response:*** *This important information, along with ascorbate concentration, has been added to the figure legend as requested.*

page 12, The table must be numbered

***Response:*** *To address this comment, the relevant table is now separated as 'Table 1'.*

page 13, figure 10: You should indicate how much DOPA was added in the culture

***Response:*** *This important information has been added to the figure legend as requested.*

Page 13, line 435 and after: an indication about the expected yield and the actual yield of THF from DOPA would be relevant, in order to better appreciate the efficiency of the synthetic pathway.

***Response:*** *The following sentence has been added to this section to address this comment:*

*'With a modest in vivo THP yield of 0.715% from 450 µM L-DOPA, many conditions must be optimized to reach our in vitro yields of 23% (219 µM) and computational yields of over 50%.'*

Page 14, line 478-479: 'high concentration '.. of DOPA not completely consumed? Do you mean that if DOPA is very low, the consumption would be complete?

***Response:*** *We did not test L-DOPA at lower than 400 µM in the current set of experiments, but it is possible that lower substrate concentration could be better for reticuline production. This should be tested in the near future, and the next sentence has been modified to include substrate concentration for improvement of the system.*

Page 18; line 642: Why do you add PLP and not simply pyridoxine to the culture? Is this addition of PLP necessary?

***Response:*** *Our best results were obtained after addition of PLP so we hesitated to stop including it in our follow up experiments. Pyridoxine should be systematically tested in the near future, together with supplementation by other cofactors to assist SAM cofactor recycling.*

Reviewer #4 (Remarks to the Author):

My specific comments have now been addressed. The dynamical modelling has been given more emphasis and the robustness has been explored and verified.

***Response:*** *We are relieved that our revisions were sufficient. Great thanks to the Reviewer for their time and effort in reviewing our manuscript.*

**Response to Reviewer Comments on:**
**Mechanism Based Tuning of Insect 3,4-Dihydroxyphenylacetaldehyde Synthase for Synthetic Bioproduction of Benzylisoquinoline Alkaloids**

## Reviewer #1 (Remarks to the Author):

This revised manuscript has been quite improved, and has included a lot more data. However, I still feel this paper is not well presented, and I am also not sure if this story is novel enough for this journal.

***Response:*** *We thank the reviewer for believing that the manuscript is improved. Great efforts have been taken to directly address all of the Reviewer's additional concerns. This includes increasing total downstream BIA over 30-fold and removal of all mention of 'DBTL'. To improve the presentation of the manuscript, it has been reorganized to focus on the big picture of developing enzyme engineering to expand the range of bioproduction targets. The use of BIA bioproduction is then presented as a concrete example of a functional enzyme engineering application. In addition, all figures have been revised to further improve the presentation.*

First, figure 1, it is still very difficult to understand the purpose of this figure. If the author wants to present this D-B-T-L as a new methodology, I won't really agree with that. Many previous stories have well demonstrated such workflow, for example, the ref 27. I don't think this is the major selling point of this story. Rather, the engineered bifunctional DHPAAS and a potential high producer of reticuline will be more appealing.

***Response:*** *To clearly address this important comment, all direct mention of 'DBTL' has been deleted from the manuscript, and Figure 1 has been totally removed.*

However, the reticuline production is ~0.2uM, which is ~ 66mg/L. The author demonstrates that this is up to date the highest, which is not true. The recent published paper on BIA analogs bi-production in E. coli (https://doi.org/10.1038/s41598-018-26306-7) has presented that production of (S)-reticuline is up to 150mg/L in the engineered E. coli strain, which should be a better comparison to this paper rather than ref 27 using yeast as the producer. 66mg/L is thus not an appealing yield to be achieved from E. coli.

***Response:*** *We agree that the reference by our co-author Dr. Minami should be carefully considered for increasing the reticuline titers. This recommended study is now cited for comparison in the discussion, and our titers are no longer compared to the previous reference 27. To thoroughly address this comment we worked very hard to increase the BIA titers. Although this request was quite challenging, we really*

*appreciate the suggestion, as the improved the BIA titers contribute to a much stronger manuscript.*

*The current study actually began in collaboration with Dr. Minami when MAO was identified as a bottleneck in his system, and this initiated our search for alternatives enzymes to MAO, leading to our engineering of DHPAAS.*

*In order to specifically address this important point, we initially constructed new combinations of DHPAAS, DDC and BIA methyltransferases, including a wild-type DHPAAS + DHPAAS-Phe79Tyr-Tyr80Phe-Asn192His system as recommended by the Reviewer below.*

*These new combinations resulted in increased production of dopamine and especially DHPAA, leading to an increase in THP titers to above 2 μM. However, simply increasing THP production did not overcome the THP to reticuline bottleneck. Analysis of the intermediates from THP to reticuline indicated that the bottleneck is pronounced at the 6-OMT step. Although there was some relative relief of the bottleneck from THP to reticuline using the Reviewer suggested combination, overall reticuline titers did not improve at this stage. These preliminary results are shown below.*

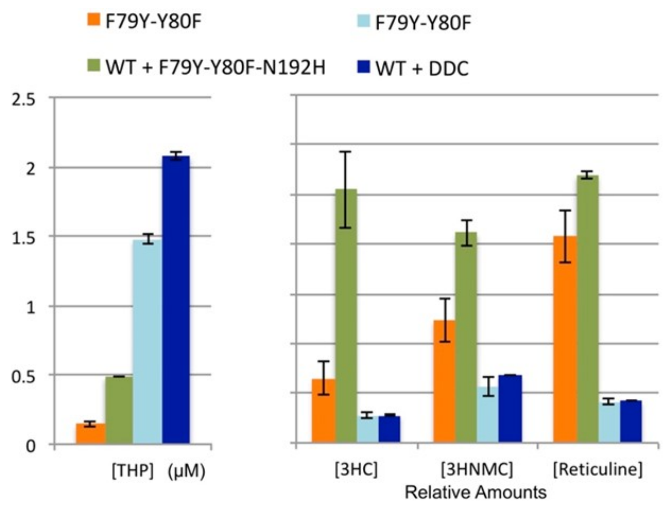


*To search for clues to overcome the THP to reticuline bottleneck, an expanded metabolomics analysis was then performed, including 115 central metabolites. Key metabolites involved in the production of reticuline using our system were identified. The related pathways are shown below with analyzed metabolites shown in bold.*

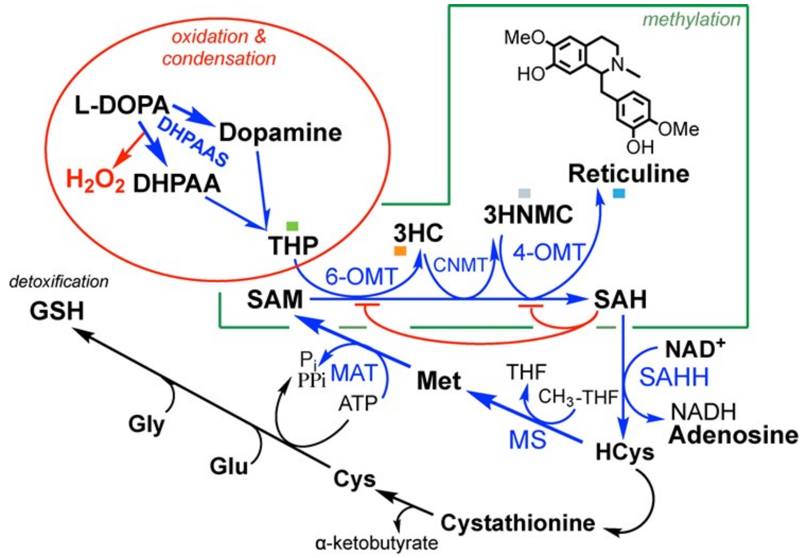


*The metabolomics analysis indicates that DHPAAS mediated THP production (within the red circle) and methylation of THP to reticuline (within the green box) must be carefully balanced. In order to increase THP production, the use of minimal medium is critical to reduce DHPAA 'drain'. In contrast, conversion of THP to reticuline is best in rich TB medium, probably due to enhanced SAM recycling. To satisfy all demands, a two-step process was modified from the original version of Dr. Minami's system, which separated THP and reticuline production (see updated references 12 and 13). The updated two-step process resulted in an increase of THP titers to 9.4 μM in the first step as shown below.*

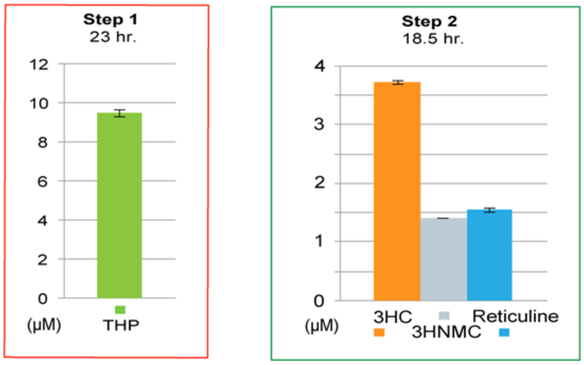


*A strain containing P. somniferum 6-OMT and CNMT was then added in the second step to help relieve the bottleneck. This enabled conversion of THP from the first step to 3.7 μM 3HC, 1.4 μM 3HNMC and 1.5 μM reticuline overnight. This represents a 7.5-fold increase in reticuline titer, and over a 30-fold increase when including all three downstream BIAs quantified in this analysis.*

*The above points demonstrate that the BIA titers of our system are scalable, directly addressing the Reviewer's concerns within the scope of this work. These important points have been incorporated into the revised manuscript.*

In addition, it seems an easy experiment for the author to coexpress the Phe79Tyr-Tyr80Phe-Asn192His variant and wildtype DHPAAS, the most efficient DHPAA and DA producing enzyme, respectively.

***Response:*** *The reviewer's recommendation is appreciated, and was helpful in improving the titers as detailed above. Accordingly BL21(DE3) containing the following genes were constructed and continuously tested during the entire revision process:*

***1)*** *3 plasmid system with wild-type DHPAAS in pTrcHis2B + DHPAAS-Phe79Tyr-Tyr80Phe-Asn192His in pE-SUMO + C. japonica 6-OMT, CNMT & 4-OMT in pACYC184.*

***2)*** *Wild-type DHPAAS in pTrcHis2B + and P. putida DDC together with the 3 C. japonica methyltransferases in pACYC184.*

***3)*** *DHPAAS-Phe79Tyr-Tyr80Phe in pTrcHis2B, and P. putida DDC together with 3 C. japonica methyltransferases in pACYC184.*

***4)*** *WT DHPAAS in pTrcHis2B + C. japonica 4-OMT, P. somniferum 6-OMT & P. somniferum CNMT in pAC23.*

***5)*** *DHPAAS-Asn192His in pTrcHis2B + C. japonica 4-OMT, P. somniferum 6-OMT & P. somniferum CNMT in pAC23.*

***6)*** *DHPAAS-Phe79Tyr-Tyr80Phe in pTrcHis2B + C. japonica 4-OMT, P. somniferum 6-OMT & P. somniferum CNMT in pAC23.*

***7)*** *DHPAAS-Phe79Tyr-Tyr80Phe-Asn192His in pTrcHis2B + C. japonica 4-OMT, P. somniferum 6-OMT & P. somniferum CNMT in pAC23.*

***8)*** *C. japonica 4-OMT, P. somniferum 6-OMT & P. somniferum CNMT in* pET23a

*Now we have also begun assembling a complete system from glucose using NCS to selectively produce (S)-reticuline to match the developed system of Dr. Minami, however we are still at the stage of optimizing tyrosine production.*

Reviewer #2 (Remarks to the Author):

The revised ms have been greatly improved and it is in a better shape for publication in Nat Comm

Yet, there is still some substantial inconsistencies or mistakes that shall be corrected before its acceptance

***Response:*** *The constructive comments are greatly appreciated, and we apologize for the inconsistencies and mistakes, which have been carefully corrected.*

page 1, line 46: I would remove this term 'Fortunately'!

***Response:*** *'Fortunately' has been removed here.*

page 2, line 59 and 63: the term 'yield ' is not used appropriately. It is rather here 'titer' or production. In addition, this titer is linked to the concentration of DOPA that is provided to the cells! Therefore, I wonder if these terms are useful!

***Response:*** *Thank you for the important comment. Improper use of 'yield' has been replaced with 'titer', 'production' and 'level' as appropriate throughout the manuscript.*

page 3, line 106: 'of' is missing at the end of the sentence

***Response:*** *We apologize for the mistake and have added the necessary 'of'.*

page 8, line 269: 'make' is missing in this sentence

***Response:*** *The reviewer must be talking in reference to the hydrogen bond. We have clarified this part by revising as: '192 residue can **form** a hydrogen bond'.*

page 11, figure 8: You should precise in the figure how much DOPA was added in the medium

***Response:*** *This important information, along with ascorbate concentration, has been added to the figure legend as requested.*

page 12, The table must be numbered

***Response:*** *To address this comment, the relevant table is now separated as 'Table 1'.*

page 13, figure 10: You should indicate how much DOPA was added in the culture

***Response:*** *This important information has been added to the figure legend as requested.*

Page 13, line 435 and after: an indication about the expected yield and the actual yield of THF from DOPA would be relevant, in order to better appreciate the efficiency of the synthetic pathway.

***Response:*** *The titer and yield of THP has improved in our new two-step experiment. Therefore, the following sentence has been added to the discussion section to address this comment:*
*'A modest cell based THP yield of 1.89% is partially due to low substrate utilization as a majority of 1 mM L-DOPA was not utilized. Many conditions must be further optimized to reach our in vitro yields of 23% (219 µM) and computational yields of over 50%.'*

Page 14, line 478-479: 'high concentration '.. of DOPA not completely consumed? Do you mean that if DOPA is very low, the consumption would be complete?
***Response:*** *As our first goal of the revision was to achieve higher tiers, we did not test* L*-DOPA at lower than 400 µM, although lower concentrations may provide higher yields. When including an additional DHPAAS or DDC, there was some improvement in the consumption of L-DOPA. To address this important point, this part of the discussion has been modified to include substrate concentration for improvement of the system.*

Page 18; line 642: Why do you add PLP and not simply pyridoxine to the culture? Is this addition of PLP necessary?
***Response:*** *The reviewers suggestion was eagerly tested, however under the conditions of this study no significant improvement was found using pyridoxine. The best results were obtained after addition of PLP so we hesitated to stop including it in our follow up experiments.*

Reviewer #4 (Remarks to the Author):

My specific comments have now been addressed. The dynamical modelling has been given more emphasis and the robustness has been explored and verified.
***Response:*** *It is a relief that our revisions were sufficient. We sincerely thank the Reviewer for their time and effort in reviewing our manuscript.*

REVIEWERS' COMMENTS:

Reviewer #1 (Remarks to the Author):

I think it is nice to discover and optimize alternative enzymes that bypass the rate limiting step by MAO, which is not as selective. However, I am getting more confused about the significance with the revisions.

First of all, I made a wrong calculation, reticuline titer in ref 32 was ~150mg/L; in this story, in the previous round was 66ug/L instead of 66mg/L; and after dedicated optimizations, it increased to 1.5uM, which is actually ~0.5mg/L. This is hundreds-of-folds of difference. If that's the case, why bypass DDC and NCS? Reticuline is the true key intermediate in opioid biosynthesis. And this story is using opioid bio-production as the basis: two out of four paragraphs in the introduction are telling about how opiod biosynthesis is important and the current strategy/enzyme is not as good. If the discovered and engineered enzyme cannot overcome MAO in opioid biosynthesis, it is not scientifically correct to make such statement that "A synthetic biology workflow was applied to engineer enzymes and pathways for the improvement of benzylisoquinoline alkaloid (BIA) biosynthesis".

Second, not sure what "a first clear example of bifunctional enzyme mechanism switching applied to synthetic biology bioproduction" means.

Reviewer #2 (Remarks to the Author):

The new version is now very good and has included all previous suggestions
still, if acceptable by the publisher, it would be worth having bigger figures or increase the front size

**REVIEWERS' COMMENTS:**

**Reviewer #1 (Remarks to the Author):**

I think it is nice to discover and optimize alternative enzymes that bypass the rate limiting step by MAO, which is not as selective. However, I am getting more confused about the significance with the revisions.

First of all, I made a wrong calculation, reticuline titer in ref 32 was ~150mg/L; in this story, in the previous round was 66ug/L instead of 66mg/L; and after dedicated optimizations, it increased to 1.5uM, which is actually ~0.5mg/L. This is hundreds-of-folds of difference. If that's the case, why bypass DDC and NCS? Reticuline is the true key intermediate in opioid biosynthesis. And this story is using opioid bio-production as the basis: two out of four paragraphs in the introduction are telling about how opiod biosynthesis is important and the current strategy/enzyme is not as good. If the discovered and engineered enzyme cannot overcome MAO in opioid biosynthesis, it is not scientifically correct to make such statement that "A synthetic biology workflow was applied to engineer enzymes and pathways for the improvement of benzylisoquinoline alkaloid (BIA) biosynthesis".

***Response:*** *We are very happy that the Reviewer thinks the selection of DHPAAS to bypass MAO is good. This important point about MAO selectivity is now emphasized in the Editor's summary. The Reviewer's comments are greatly appreciated, and we thank the reviewer again for their encouragement as it led to significant improvements in the manuscript.*

*To address the additional concerns, we first reduced descriptions of opioids to a single paragraph in the introduction.*

*We apologize for the confusing statement in regards to the titers, and have deleted the mentioned statement about the "synthetic biology workflow" from the revised abstract. Furthermore, we now only present the DHPAAS-mediated pathway as an alternative to MAO, while clarifying that further improvements to BIA production are required in future studies.*

*These important revisions are also further emphasized in the revised discussion and in the above response to the Editorial Requests.*

Second, not sure what "a first clear example of bifunctional enzyme mechanism switching applied to synthetic biology bioproduction" means.

***Response:*** *We apologize for this vague statement. To clarify:*

*"a first clear example of bifunctional enzyme mechanism switching applied to synthetic biology bioproduction", has been revised as:*

*“the current study provides an example of functional enzyme engineering applied to an alternative bioproduction pathway.”*

**Reviewer #2 (Remarks to the Author):**

The new version is now very good and has included all previous suggestions still, if acceptable by the publisher, it would be worth having bigger figures or increase the front size

***Response:*** *Thank you for believing the revisions are good. Font size has been increased in revised Figures 1, 2, 3, 4, 5, 6, 7, 8 and 9. Original .ai and .pptx files are also included in case editorial formatting is needed.*
